# Treatment of advanced BP-NETS with lanreotide autogel/depot vs placebo: the phase III SPINET study

**DOI:** 10.1530/ERC-23-0337

**Published:** 2024-07-22

**Authors:** E Baudin, J Capdevila, D Hörsch, S Singh, M E Caplin, E M Wolin, W Buikhuisen, M Raderer, E Dansin, C Grohe, D Ferone, A Houchard, X-M Truong-Thanh, D Reidy-Lagunes, the SPINET Study Group

**Affiliations:** 1 Endocrine Oncology Unit, Imaging Department, Gustave Roussy, Villejuif, France; 2 Medical Oncology Department, Vall d’Hebron University Hospital, Vall d’Hebron Institute of Oncology (VHIO), IOB Quirón-Teknon, Barcelona, Spain; 3 ENETS Center of Excellence, Zentralklinik Bad Berka GmbH, Bad Berka, Germany; 4 Division of Medical Oncology, University of Toronto, Sunnybrook Odette Cancer Center, Sunnybrook HSC, Toronto, Ontario, Canada; 5 Neuroendocrine Tumour Unit, Royal Free Hospital School of Medicine, London, UK; 6 Division of Hematology and Oncology, Icahn School of Medicine at Mount Sinai, New York, New York, USA; 7 Department of Thorax Oncology, Netherlands Cancer Institute, Amsterdam, the Netherlands; 8 Division of Oncology, Department of Medicine I, Medical University of Vienna, Vienna, Austria; 9 Thoracic Oncology Unit, Centre Oscar Lambret, Lille, France; 10 Department of Respiratory Diseases, Evangelische Lungenklinik, Berlin, Germany; 11 Neuroendocrine Tumour Unit, Department of Internal Medicine and Medical Specialties, University of Genova, Genova, Italy; 12 Data and Insights Generation and Strategy, Ipsen, Boulogne-Billancourt, France; 13 Medical Affairs, Ipsen, Boulogne-Billancourt, France; 14 Department of Medicine, Memorial Sloan Kettering Cancer Center, Weill Cornell Medical Center, New York, New York, USA

**Keywords:** atypical carcinoid, bronchopulmonary neuroendocrine tumors, somatostatin analog, typical carcinoid

## Abstract

Prospective data are lacking on early somatostatin analog (SSA) therapy in bronchopulmonary neuroendocrine tumors (BP-NETs; typical carcinoids and atypical carcinoids (TCs and ACs)). SPINET (EudraCT: 2015-004992-62; NCT02683941) was a phase III, double-blind study of lanreotide autogel/depot (LAN; 120 mg every 28 days) plus best supportive care (BSC) vs placebo plus BSC, with an optional open-label treatment phase (LAN plus BSC). Patients had metastatic/unresectable, somatostatin receptor (SSTR)-positive TCs or ACs. Recruitment was stopped early owing to slow accrual; eligible patients from the double-blind phase transitioned to open-label LAN. The adapted primary endpoint was progression-free survival (PFS) during either phase for patients receiving LAN. Seventy-seven patients were randomized (LAN, *n* = 51 (TCs, *n* = 29; ACs,* n* = 22); placebo, *n* = 26 (TCs, *n* = 16; ACs, *n* = 10)). Median (95% CI) PFS during double-blind and open-label phases in patients receiving LAN was 16.6 (11.3; 21.9) months overall (primary endpoint), 21.9 (12.8, not calculable (NC)) months in TCs, and 13.8 (5.4; 16.6) months in ACs. During double-blind treatment, median (95% CI) PFS was 16.6 (11.3; 21.9) months for LAN vs 13.6 (8.3; NC) months for placebo (not significant); corresponding values were 21.9 (13.8; NC) and 13.9 (13.4; NC) months, respectively, in TCs and 13.8 (5.4; 16.6) and 11.0 (2.8; 16.9) months, respectively, in ACs. Patients’ quality of life did not deteriorate and LAN was well tolerated. Although recruitment stopped early and the predefined sample size was not met, SPINET is the largest prospective study to date of SSA therapy in SSTR-positive TCs and ACs and suggests clinical benefit in TCs.

## Introduction

Bronchopulmonary (BP) neuroendocrine tumors (NETs; typical carcinoids and atypical carcinoids (TCs and ACs)) account for 20–25% of NETs and 1–2% of lung malignancies (Öberg *et al.* 2012, Filosso *et al.* 2018, Baudin *et al.* 2021). In patients with metastatic TCs or ACs, median overall survival was reported to be 80.9 months, ranging from 33 to 105 months according to prognostic factors (Robelin *et al.* 2019).

Many NETs overexpress somatostatin receptors (SSTRs), leading to the development of somatostatin analogs (SSAs) as antisecretory and antiproliferative treatments across different NET primaries (Del Olmo-Garcia *et al.* 2021). Two SSAs have been shown to significantly delay progression or death in metastatic gastrointestinal NETs: lanreotide autogel/depot (LAN) in patients with slowly progressive well- or moderately differentiated gastroenteropancreatic (GEP) NETs (Caplin *et al.* 2014), and octreotide long-acting release (OCT) in patients with well-differentiated midgut NETs and a mainly low hepatic tumor burden (Rinke *et al.* 2009). SSAs also have favorable tolerability profiles and are the initial mainstay of medical treatment for gastrointestinal NETs with a good prognosis.

TCs and ACs express SSTRs, providing a rationale for their treatment with SSAs (Vesterinen *et al.* 2019). However, when the current study (SPINET) was designed, the data on the use of SSAs in patients with these tumors were limited to those from a small subgroup (*n* = 11) of the RADIANT-2 study (Pavel *et al.* 2011) and retrospective analyses (Lopez *et al.* 2014, Karra *et al.*2015, Sullivan *et al.* 2015), and LAN was not yet recommended as an antiproliferative treatment for TCs and ACs in US and European guidelines (Öberg *et al.* 2012, Kunz *et al.* 2013). The SPINET study was designed to address this unmet need by prospectively evaluating the efficacy and safety of LAN in patients with SSTR-positive TCs and ACs.

## Materials and methods

Enrollment began in July 2016. In July 2018, recruitment was stopped owing to slow accrual, resulting primarily from changes to US and EU guidelines. The protocol was amended, including the primary objective and primary and secondary endpoints (see below). Eligible patients still receiving treatment during the double-blind phase could enter the open-label treatment phase to receive open-label LAN.

### Patients

Full inclusion and exclusion criteria are summarized in Supplementary Table S1 (see section on supplementary materials given at the end of this article). Patients, who were enrolled by study investigators at specialist centers, were eligible if they were aged ≥ 18 years and had metastatic and/or unresectable, pathologically confirmed TCs or ACs with positive SSTR imaging. They also needed at least one measurable lesion of the disease on CT or MRI (Response Evaluation Criteria in Solid Tumors (RECIST) v1.1 (Eisenhauer *et al.* 2009)) and an Eastern Cooperative Oncology Group (ECOG) performance status score of 0 or 1. Patients were excluded if they had received previous SSA treatment, more than two lines of chemotherapy (cytotoxic, molecular targeted therapy, or interferon-alpha) for TCs/ACs, or chemotherapy in the 4 weeks before randomization, or if they had functional disease requiring SSA treatment for symptom management. No assessment of disease progression was performed before study entry (not an inclusion criterion).

### Study design and interventions

SPINET (EudraCT: 2015-004992-62; NCT02683941) was a phase III, prospective, multicenter, randomized, double-blind, placebo-controlled study with an optional open-label LAN treatment phase and an open-label follow-up phase. Patients were randomly allocated (2:1) to receive subcutaneous injections every 28 days with LAN (120 mg) plus best supportive care (BSC) or placebo plus BSC, with randomization stratified by TC vs AC and prior vs no prior chemotherapy (see Supplementary Material for information about randomization and blinding).

The plan was to enroll 216 patients across Canada, Europe, and the USA, but recruitment was stopped early owing to slow accrual. Before recruitment was stopped, patients receiving a placebo with centrally confirmed progression (RECIST v1.1 (Eisenhauer *et al.* 2009)) during the double-blind phase could enter the open-label treatment phase to receive LAN plus BSC. After recruitment was stopped, eligible patients remaining in the double-blind phase (i.e. patients receiving LAN without centrally confirmed disease progression and patients receiving placebo) could transition to open-label LAN + BSC.

The study ended 18 months after the last patient was randomized, in February 2020.

### Assessments and endpoints

To assess disease progression, patients underwent a CT or MRI scan at baseline and every 12 weeks during the study. Procedures for local and central review are summarized in Supplementary Fig. S1. If locally assessed disease progression was not confirmed centrally, the local decision prevailed, in accordance with US Food and Drug Administration requirements. Other assessments during the study are summarized in Supplementary Tables S2 and S3.

Some endpoints were amended after recruitment was stopped. The primary endpoint was changed from centrally confirmed progression-free survival (PFS) during the double-blind phase to centrally confirmed PFS during the double-blind or open-label treatment phases for patients randomized to LAN only; PFS endpoints for LAN vs placebo became secondary endpoints. Key secondary endpoints, assessed for the LAN and placebo groups during the double-blind phase, were centrally and locally assessed PFS, objective response rate (ORR), and time to treatment failure (TTF). Other secondary endpoints, assessed during the double-blind and open-label treatment phases, included changes in the European Organization for Research and Treatment of Cancer (EORTC) Quality of Life Questionnaire Core 30 (QLQ-C30), proportions of patients with a deterioration in QLQ-C30 scores (≥ 10-point decrease), and changes in biomarker levels (serum chromogranin (CgA) and urinary 5-hydroxyindoleacetic acid (5-HIAA); see Supplementary Material). Exploratory endpoints included clinical benefit rate (CBR), defined as the proportion of patients with complete response, partial response, or stable disease, and tumor growth rate (TGR) during the double-blind and open-label treatment phases. Safety endpoints were treatment-emergent adverse events (TEAEs), laboratory parameters (hematology and biochemistry), vital signs, and ECG results. Patients had gallbladder echography if biological abnormalities and/or clinical inflammatory symptoms occurred during the study.

### Statistical analysis

The planned sample size was 216 patients (LAN, *n* = 144; placebo, *n* = 72; i.e. 2:1 ratio), based on 90% power to detect a clinically meaningful treatment difference of 4 months for PFS (10 months for LAN and 6 months for placebo; i.e. an expected hazard ratio (HR) of 0.6), with a type 1 error rate of 0.05.

The intention-to-treat (ITT) population comprised all patients randomly assigned to study treatment. The open-label ITT population comprised all ITT patients who entered the open-label treatment phase and received at least one LAN injection during this phase. The safety population included all patients who received at least one injection of study medication.

PFS and TTF were analyzed using the Kaplan–Meier product limit method. Associated HRs were estimated using a stratified Cox proportional hazards model, and 95% CIs were calculated using the Brookmeyer–Crowley method. For ORR, CBR, and the proportions of patients with decreases in biomarker CgA and QLQ-C30 scores, 95% CIs were calculated using the Clopper–Pearson method. Statistical significance was assessed using a stratified log-rank test.

TGR was calculated from radiologic scans in the 12 months before the study, at baseline, and every 12 weeks during the study, and estimated using the sum of diameters of target lesions (100 (exp(3 log (D*t*/D0)/*t*) − 1)) (D0 and D*t* = tumor sizes at times 0 and *t*). Given that the provision of historical scans was optional, TGR at baseline could only be computed for the subgroup of patients for whom historical scans were available.

Statistical analyses were performed with SAS^®^ software, v9.4 or higher (SAS Institute, Inc.).

### Ethical approval

The study was conducted in compliance with the Declaration of Helsinki, the International Conference on Harmonization Good Clinical Practice guidelines, and institutional review board/independent ethics committee requirements. All patients had to provide written informed consent before enrollment.

## Results

### Patients

**Figure 1:**
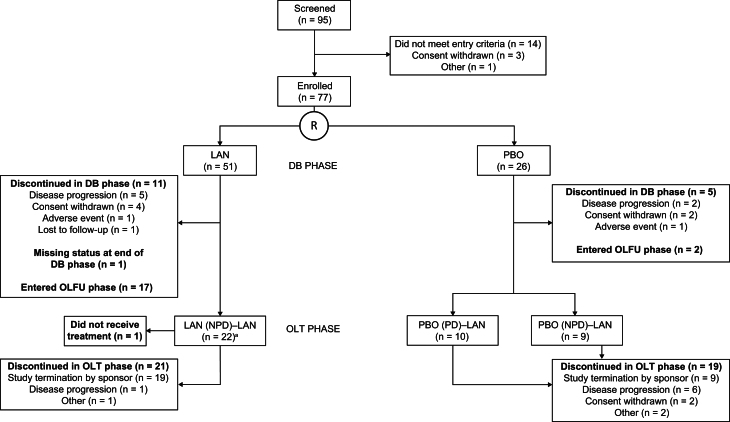
Patient disposition. Data from the OLFU phase are not reported in this article; the group comprised 19 patients who transitioned from the DB phase (as noted in the figure above) and one patient from the LAN (NPD)–LAN group who entered OLFU from the OLT phase. ^a^All 22 patients transitioned to the OLT phase after protocol amendment #5. DB, double-blind; LAN, lanreotide autogel/depot; NPD, no progressive disease; OLFU, open-label follow-up; OLT, open-label treatment; PBO, placebo; PD, progressive disease; R, randomization.

Overall, 77 patients were enrolled (51 randomized to LAN, 26 to placebo) (Fig. 1); 21 in the LAN group and 19 in the placebo group received LAN during the open-label treatment phase (Fig. 1). Given that recruitment was stopped prematurely, the planned sample size was not met, and statistical analyses are considered descriptive. The median (range) on-study duration was 13.1 (1–34) months (double-blind phase), 8.1 (0–21) months (open-label treatment phase), and 22.8 (2–36) months (overall). Five patients (9.8%) in the LAN group and four patients (15.4%) in the placebo group had received prior systemic therapy (Table 1).

**Table 1:** Baseline demography and clinical characteristics (ITT population).

	**LAN (*n* = 51**	Placebo (*n* = 26)
Age (years), mean (s.d.)	66.4 (11.9)	65.8 (13.9)
Male, *n* (%)	28 (54.9)	14 (53.8)
Race, *n* (%)^a^	*n* = 39	*n* = 19
White	35 (89.7)	19 (100)
Black or African American	1 (2.6)	0
Asian	3 (7.7)	0
Time since diagnosis (months), median (95% CI)^b^	10.0 (5.1; 19.2)	5.4 (3.1; 31.8)
NET type, *n* (%)^c^		
Typical	29 (56.9)	16 (61.5)
Atypical	22 (43.1)	10 (38.5)
Mitotic count, *n* (%)^d^		
<2 mitoses/2 mm^2^	32 (62.7)	17 (65.4)
2–10 mitoses/2 mm^2^	19 (37.3)	9 (34.6)
Foci of necrosis, *n* (%)^d^		
Absent	42 (82.4)	24 (92.3)
Present	9 (17.6)	2 (7.7)
Proliferation index Ki-67, *n* (%)^e^		
<10%	32 (62.7)	16 (61.5)
≥10 to <20%	10 (19.6)	5 (19.2)
≥20%	6 (11.8)	2 (7.7)
Missing	3 (5.9)	3 (11.5)
ECOG performance status, *n* (%)		
0	34 (66.7)	14 (53.8)
1	17 (33.3)	12 (46.2)
Bone metastases, *n* (%)	13 (25.5)	6 (23.1)
Hepatic tumor load, *n* (%)		
≤25%	47 (92.2)	24 (92.3)
>25%	2 (3.9)	0
Missing	2 (3.9)	2 (7.7)
Octreoscan/Krenning scale score, *n* (%)^f^		
2	9 (30.0)	5 (41.7)
3	15 (50.0)	5 (41.7)
4	6 (20.0)	2 (16.7)
Uptake greater than background liver, *n* (%)^g^		
Yes	20 (95.2)	13 (92.9)
No	0	1 (2.9)
Missing	1 (4.8)	0
Previous treatment for BP-NET, *n* (%)		
Surgery	29 (56.9)	12 (46.2)
Somatostatin analogs^h^	2 (3.9)	0
Everolimus^i^	1 (2.0)	0
Cytotoxic chemotherapy^j,k^	5 (9.8)	4 (15.4)
Radiotherapy	6 (11.8)	6 (23.1)

Data are from the baseline of the double-blind phase.

^a^It was not permitted to collect race data in France and Poland; ^b^Data missing for three patients in each group; ^c^According to interactive web response system; ^d^Obtained as part of disease history/diagnosis at screening; ^e^Obtained as part of disease history/diagnosis at screening, if available; ^f^Percentages based on the total number of patients with Octreoscan results; ^g^Percentages based on the total number of patients with positron emission tomography scans; ^h^Lanreotide (*n* = 1), octreotide (*n* = 1); ^i^This patient also received cytotoxic chemotherapy; ^j^These comprised etoposide (*n* = 3), carboplatin (*n* = 2), cisplatin (*n* = 4), capecitabine (*n* = 1), temozolomide (*n* = 2), vinorelbine (*n* = 1) in the LAN group, and carboplatin (*n* = 1), cisplatin (*n* = 3), etoposide (*n* = 3), paclitaxel (*n* = 1), pemetrexed (*n* = 1), and vincristine (*n* = 1) in the placebo group; ^k^According to electronic case report form.

BP, bronchopulmonary; ECOG, Eastern Cooperative Oncology Group; ITT, intention-to-treat; LAN, lanreotide autogel/depot; NET, neuroendocrine tumor.

Overall, the mean (s.d.) age was 66.2 (12.5) years, and 54.5% of patients were male. Among them, 58.4% had TCs (29 patients in the LAN arm and 16 patients in the placebo arm) and 41.6% had ACs (22 patients in the LAN arm and 10 patients in the placebo arm (Table 1)). There were some imbalances in baseline characteristics between the two groups, including the median time since diagnosis and the proportion of patients with ECOG performance status 0 or 1 (Table 1 and Supplementary Table S4).

### Efficacy

#### Primary endpoint

**Figure 2:**
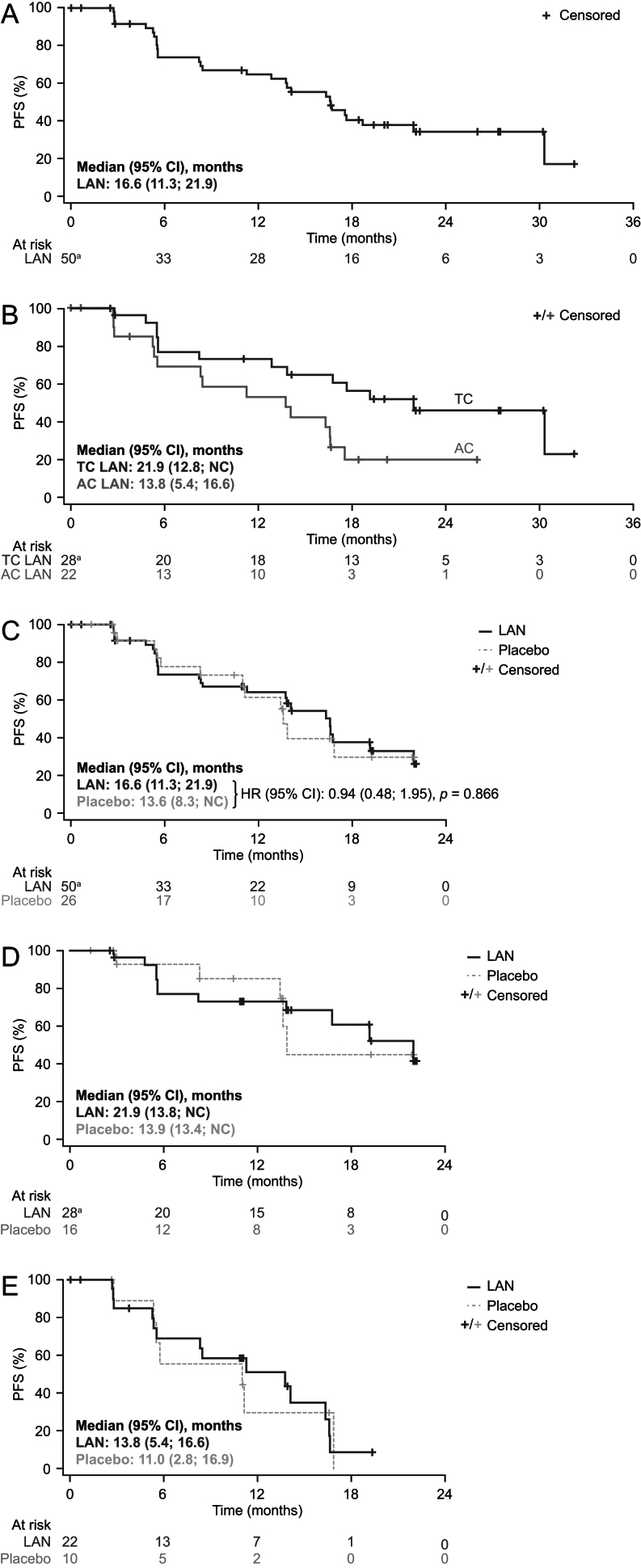
Centrally assessed (RECIST v1.1) progression-free survival (ITT population). (A) During double-blind or open-label treatment phase in patients randomly assigned to LAN (overall patient population). (B) During the double-blind or open-label treatment phase in patients randomly assigned to LAN (by tumor subtype). (C) During the double-blind phase in patients randomly assigned to LAN or placebo (overall patient population). (D) During the double-blind phase in patients randomly assigned to LAN or placebo (in patients with typical carcinoids). (E) During the double-blind phase in patients randomly assigned to LAN or placebo (in patients with atypical carcinoids). Data from patients who did not die and did not have confirmed disease progression were censored on the date of the last radiological assessment at which the target lesions were evaluated by central review. ^a^One patient was excluded from the analysis because their data were censored at baseline (because the baseline assessment was prior to randomization, this would have yielded a negative PFS). PFS in months was calculated as follows: (date of event – date of randomization) / 30.4375. AC, atypical carcinoid; HR, hazard ratio; ITT, intention-to-treat; LAN, lanreotide autogel/depot; NC, not calculable; PFS, progression-free survival; RECIST, Response Evaluation Criteria in Solid Tumors; TC, typical carcinoid.

The median (95% CI) centrally assessed PFS in LAN-randomized patients during the double-blind and open-label treatment phases was 16.6 (11.3; 21.9) months (Fig. 2A and Supplementary Table S5). In subgroups of patients with TCs and ACs, the median (95% CI) centrally assessed PFS during the double-blind and open-label phases was 21.9 (12.8; not calculable (NC)) and 13.8 (5.4; 16.6) months, respectively (Fig. 2B).

#### Secondary and exploratory endpoints

During the double-blind phase, the median (95% CI) centrally assessed PFS was 16.6 (11.3; 21.9) months for LAN vs 13.6 (8.3; NC) months for placebo, but the difference was not statistically significant (HR 0.94 (95% CI 0.48; 1.95); *P* = 0.866) (Fig. 2C). The median (95% CI) centrally assessed PFS in patients with TCs was 21.9 (13.8; NC) months for LAN and 13.9 (13.4; NC) months for placebo (Fig. 2D); the median (95% CI) centrally assessed PFS in patients with ACs was 13.8 (5.4; 16.6) months and 11.0 (2.8; 16.9) months, respectively (Fig. 2E). PFS based on local assessments is summarized in Supplementary Table S6.

**Figure 3:**
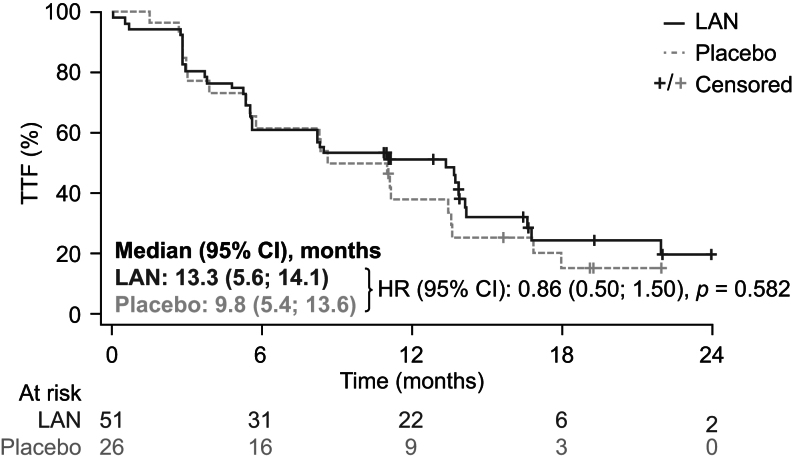
Time to treatment failure during the double-blind phase (ITT population). TTF was defined as the time from randomization to: documented central or local PD (defined as the minimum time to event according to central/local review using RECIST v1.1); treatment discontinuation for undocumented disease progression; treatment discontinuation for toxicity or any other reason; start of new anticancer treatment; withdrawal of consent; an AE; a protocol deviation; loss to follow-up; appearance of carcinoid syndrome or other hormone-related syndrome necessitating the initiation of SSAs (rescue octreotide and/or long-acting release SSA); death between adequate assessment visits; death before first PD assessment; prohibited medication/therapy or unblinded event. TTF in months was calculated as follows: (date of event − date of randomization)/30.4375. AE, adverse event; HR, hazard ratio; ITT, intention-to-treat; LAN, lanreotide autogel/depot; PD, progressive disease; RECIST, Response Evaluation Criteria in Solid Tumors; SSA, somatostatin analog; TTF, time to treatment failure.

During the double-blind phase, the centrally assessed ORR was 14.0% for LAN and 0% for placebo, but the difference was not statistically significant (Table 2). CBR was similar (90.0% vs 92.0%) between the two groups (Table 2). TTF (median (95% CI)) was 13.3 (5.6; 14.1) months for LAN vs 9.8 (5.4; 13.6) months for placebo (HR 0.86 (95% CI, 0.50; 1.50)), but this difference was not statistically significant (*P* = 0.582) (Fig. 3); see Supplementary Table S7 for reasons for treatment failure. The ORR for LAN was 6.0% on local assessment and 14.0% on central assessment. The ORR and CBR based on local assessments are summarized in Supplementary Table S8 and further information provided in the Supplementary Material.

**Table 2:** Secondary efficacy endpoints: objective response rate^a^ and clinical benefit rate^b^ (centrally assessed; ITT population).

Double-blind phase							
	*n* (%) (95% CI)					LAN vs PBO treatment difference (95% CI)	
	**LAN (*****n***** = 51)**		**PBO (*****n*****= 26)**		
ORR^a^	7/50 (14.0) (5.82; 26.74)		0/25 (0) (0.00; 13.72)			14.0 (−10.97; 37.86)	
CBR^b^	45/50 (90.0) (78.19; 96.67)		23/25 (92.0) (73.97; 99.02)			−2.00 (−26.53; 22.69)	
**Open-label treatment phase**							
	***n***** (%) (95% CI)**					**Treatment difference (95% CI)**	
	LAN (NPD)–LAN (***n***** = 21)**	PBO (NPD)–LAN (***n***** = 9)**		PBO (PD)–LAN (***n*****= 10)**	All patients (***n***** = 40)**	LAN (NPD)–LAN vs PBO (NPD)–LAN	LAN (NPD)–LAN vs PBO (PD)–LAN
CBR^b^	21/21 (100) (83.89; 100)	8/8 (100) (63.06; 100)		7/9 (77.8) (39.99; 97.19)	36/38 (94.7) (82.25; 99.36)	0 (NC; NC)	22.2 (−17.64; 60.01)

Note that ORR during the open-label treatment phase is not provided because it was not a pre-specified endpoint. Tumors were assessed according to RECIST v1.1 criteria.

^a^Complete responses plus partial responses; ^b^Complete responses plus partial responses plus stable disease.

CBR, clinical benefit rate; ITT, intention-to-treat; LAN, lanreotide autogel/depot; NC, not calculable; NPD, no progressive disease; ORR, objective response rate; PBO, placebo; PD, progressive disease; RECIST, Response Evaluation Criteria in Solid Tumors.

EORTC QLQ-C30 scores during the double-blind phase and open-label phase are shown in Supplementary Figs S2 and S3. In the double-blind phase, EORTC QLQ-C30 scores (Supplementary Fig. S2) were similar between the LAN and placebo groups; for example, at week 48, the mean (95% CI) changes were −6.3 (−12.6; −0.1) and −4.2 (−18.2; 9.8) for global health status scores, respectively. The proportions of patients with a ≥ 10-point decrease (deterioration) in QLQ-C30 global scores were broadly similar in the LAN and placebo groups (Supplementary Table S9).

Changes in serum CgA and urinary 5-HIAA levels during the double-blind phase are shown in Supplementary Figs S4 and S5, respectively. In patients with elevated CgA levels at baseline (≥ 2 × upper limit of normal; *n* = 30 in the LAN group and *n* = 13 in the placebo group), the proportions with a ≥ 30% reduction in CgA at week 8 were 63.3% and 7.7%, respectively.

At baseline, the mean (95% CI) TGR was 14.2% (3.86; 24.49%) per month for LAN (*n* = 15) and 2.6% (−8.11; 13.32%) per month for placebo (*n* = 7). At week 12, the mean (95% CI) change from baseline (%/month) in TGR was −11.8% (−22.51; −1.15%) and +11.0% (−3.99; 26.00%), respectively. Results obtained during the remainder of the double-blind phase are summarized in Supplementary Table S10.

### Safety

During the double-blind phase, TEAEs were reported for similar proportions of patients in the LAN and placebo groups (96.1% and 96.2%, respectively); in most cases, the events were of grade 1 or 2 severity (Table 3). TEAEs leading to treatment discontinuation were reported in two patients (3.9%) receiving LAN and three patients (11.5%) receiving placebo (Table 3). Serious TEAEs were reported in ten patients (19.6%) and seven patients (26.9%), respectively (treatment-related in 2 (3.9%) and 1 (3.8%) cases). Treatment-related TEAEs during the double-blind phase were reported in 74.5% of patients receiving LAN and in 53.8% of those receiving placebo. The most common treatment-related TEAEs in the LAN group were diarrhea (52.9%; 15.4% for placebo), upper abdominal pain/abdominal pain (11.8%; 15.4% for placebo), fatigue (11.8%; 15.4% for placebo), and flatulence (11.8%; 3.8% for placebo) (Supplementary Table S11).

**Table 3:** Safety profile (safety and open-label ITT populations).

	*n* (%)				
	Double-blind phase		Open-label treatment phase		
	LAN group (*n* = 51)	PBO group (*n* = 26)	LAN (NPD)–LAN group (*n* = 21)	PBO (NPD)–LAN group (*n* = 9)	PBO (PD)–LAN group (*n* = 10)
Any TEAE	49 (96.1)	25 (96.2)	9 (42.9)	8 (88.9)	9 (90.0)
Grade 1	44 (86.3)	23 (88.5)	8 (38.1)	8 (88.9)	9 (90.0)
Grade 2	37 (72.5)	19 (73.1)	6 (28.6)	2 (22.2)	6 (60.0)
Grade 3	13 (25.5)	8 (30.8)	1 (4.8)	1 (11.1)	1 (10.0)
Grade 4	1 (2.0)	0	0	0	0
Grade 5	1 (2.0)	0	0	0	0
Any TEAE related to treatment	38 (74.5)	14 (53.8)	3 (14.3)	4 (44.4)	6 (60.0)
Grade 1	31 (60.8)	13 (50.0)	3 (14.3)	4 (44.4)	5 (50.0)
Grade 2	23 (45.1)	4 (15.4)	2 (9.5)	0	2 (20.0)
Grade 3	2 (3.9)	1 (3.8)	1 (4.8)	0	1 (10.0)
Grade 4	1 (2.0)	0	0	0	0
Grade 5	0	0	0	0	0
TEAEs leading to treatment discontinuation^a^	2 (3.9)	3 (11.5)	0	0	0
Serious TEAEs	10 (19.6)	7 (26.9)	0	1 (11.1)	0
Treatment-related	2 (3.9)	1 (3.8)	0	0	0
Most common^b^ TEAEs					
Diarrhea	32 (62.7)	8 (30.8)	2 (9.5)	2 (22.2)	5 (50.0)
Fatigue	13 (25.5)	10 (38.5)	0	0	1 (10.0)
Abdominal pain^c^	11 (21.6)	6 (23.1)	1 (4.8)	0	3 (30.0)
Asthenia	11 (21.6)	5 (19.2)	1 (4.8)	0	1 (10.0)
Constipation	8 (15.7)	4 (15.4)	0	0	1 (10.0)
Dizziness	8 (15.7)	2 (7.7)	0	0	2 (20.0)
Decreased appetite	6 (11.8)	6 (23.1)	2 (9.5)	0	1 (10.0)
Dyspnea	6 (11.8)	4 (15.4)	1 (4.8)	0	1 (10.0)
Nausea	6 (11.8)	4 (15.4)	2 (9.5)	0	1 (10.0)
Headache	4 (7.8)	4 (15.4)	0	0	4 (40.0)
Hyperglycemia	4 (7.8)	4 (15.4)	0	1 (11.1)	1 (10.0)
Nasopharyngitis	4 (7.8)	4 (15.4)	1 (4.8)	0	1 (10.0)
Type 2 diabetes	1 (2.0)	0	1 (4.8)	2 (22.2)	0
Tumor pain	0	0	0	0	2 (20.0)

Median (range) duration of treatment exposure during the double-blind phase was 12.9 (1–33) months in the LAN group and 11.6 (1–24) months in the PBO group; during the open-label treatment phase, it was 7.5 (5–9) months in the LAN (NPD)–LAN group, 8.4 (3–12) months in the PBO (NPD)–LAN group, and 13.5 (1–21) months in the PBO (PD)–LAN group.

Data from the double-blind phase are from the safety population and those from the open-label treatment phase are from the open-label ITT population.

^a^Carcinoid syndrome (*n* = 1) and *Pneumocystis jirovecii* infection in the LAN group, and abdominal pain (*n* = 1), musculoskeletal chest pain (*n* = 1), and pneumonia (*n* = 1) in the placebo group; ^b^Incidence ≥15% in any treatment group; ^c^Preferred terms of abdominal pain or upper abdominal pain have been combined.

ITT, intention-to-treat; LAN, lanreotide autogel/depot; NPD, no progressive disease; PBO, placebo; PD, progressive disease; TEAE, treatment-emergent adverse event.

During the open-label treatment phase, TEAEs, irrespective of causality, were reported in 88.9% of patients in the placebo (no progressive disease [NPD])–LAN group, 90.0% in the placebo (progressive disease)–LAN group, and 42.9% in the LAN (NPD)–LAN group; treatment-related TEAEs were reported in 44.4%, 60.0%, and 14.3% of patients, respectively (Table 3). There were no TEAEs leading to treatment discontinuation, and only one serious TEAE (hyperglycemia, not related to treatment) was reported.

Compared with baseline, there were no clinically relevant changes in mean values for hematologic, biochemical, or ECG parameters, estimated glomerular filtration rate, or vital signs during the double-blind and open-label treatment phases. Only two patients receiving LAN required post-baseline gallbladder echography, and neither developed new abnormalities during the study (Supplementary Material).

## Discussion

SPINET is the first phase III, randomized, placebo-controlled study to evaluate early SSA therapy in patients with TCs and ACs. It provides clinically important evidence regarding the use of LAN in patients with these tumors, for whom few treatment options are available. Although recruitment was stopped prematurely and the predefined sample size was not met, the combined results of the primary, secondary, and exploratory endpoints suggest that LAN has clinical activity, most obviously in the medically important subgroup with TCs, where the median numerical improvement in centrally assessed PFS vs placebo was 8 months. Indeed, the 21.9-month PFS for LAN in patients with TCs reported in SPINET falls within the range of 14.3 months (time to progression (TTP)) to 38.5 months (PFS) reported for OCT in the PROMID trial (patients with metastatic midgut NETs) or LAN in the CLARINET trial (patients with advanced enteropancreatic NETs), respectively (Rinke *et al.* 2009, Caplin *et al.*). The PFS with placebo in SPINET (13.9 months) is also within the range reported for placebo in the PROMID (TTP, 6.0 months) and CLARINET (PFS, 18.0 months) trials (Rinke *et al.* 2009, Caplin *et al.* 2014). In addition to the smaller-than-planned sample size, factors that may have limited the extent of the difference between treatment arms include tumor progression before enrollment not being a prerequisite (as in the PROMID trial (Rinke *et al.* 2009)), and the potentially higher TGR in the LAN arm. In addition to the numerical improvement in PFS, there were greater reductions in serum CgA and in TGR in the LAN arm. TGR, a predictive marker for progression and survival, is useful for assessing slow-growing tumors like NETs, and it has previously been shown that LAN significantly decreased TGR in patients with GEP-NETs (Dromain *et al.* 2021). Patients’ health-related quality of life did not deteriorate during treatment in the present study, and LAN was well tolerated, having a safety profile consistent with that well established in NETs (Bianchi *et al.* 2011, Martín-Richard *et al.* 2013, Caplin *et al.* 2014, Michael *et al.* 2017, Godara *et al.* 2019, Caplin *et al.* 2021, Ito *et al.* 2021, Rinke *et al.* 2021).

The main reason for the slow accrual of patients into SPINET was the increasing use of SSAs for TCs and ACs in clinical practice; as such, patients were reluctant to enroll in this study, with the risk of receiving a placebo, when they could access the active treatment with a prescription. The increase in SSA use probably reflects changes to guidelines (Caplin *et al.* 2015, Kulke *et al.* 2015), which were updated to recommend the use of SSAs/LAN as first-line antiproliferative treatment in SSTR-positive TCs and ACs to discourage the early use of platinum-based chemotherapy. At the time the guidelines changed, recommendations were based mainly on the extrapolation of findings from research in GEP-NETs (LAN) (Caplin *et al.* 2014) or midgut NETs (OCT) studies (Rinke *et al.* 2009), as well as limited data on the use of SSAs for TCs and ACs from a subgroup analysis of the RADIANT-2 study (OCT vs everolimus plus OCT) (Pavel *et al.* 2011) and three retrospective studies published as abstracts (Lopez *et al.* 2014 , Karra *et al.* 2015 , Sullivan *et al.* 2015).

After SPINET was initiated, data from a phase II study (LUNA) of SSA therapy (which included patients with progressive TCs and ACs) were published, providing the first prospective signal that SSAs (as well as everolimus alone and everolimus plus SSA) may have antiproliferative effects in these patients (Ferolla *et al.* 2017). However, LUNA was conducted in patients receiving mainly post-first-line therapy with progressive tumors according to RECIST v1.1 and included a small number of patients with thymic NETs, so its data are not directly comparable with SPINET data. Since then, the first data on cytotoxic chemotherapy, from the prospective, phase II ATLANT study (LAN plus temozolomide; *n* = 40), have been published (Ferolla *et al.* 2023). ATLANT included patients with progressive TCs (20.0%), ACs (52.5%), or unspecified carcinoids (27.5%) who had received ≤1 line of treatment with SSA. The study demonstrated an investigator-assessed disease control rate (DCR) at 9 months (primary endpoint) of 35% (partial response, 2.5%); however, this was not statistically significant at a 30% threshold (*P* = 0.2968) and the primary endpoint was not met. Median PFS was 37.1 weeks, and 32.5% of patients experienced TEAEs that led to the interruption of study medication. In a sensitivity analysis conducted using investigator assessments between months 7.5 and 10.5, DCR was 45.0% (partial response, 7.5%), which was statistically significant at a 30% threshold (*P* = 0.0320) (Ferolla *et al.* 2023). From a clinical perspective, these results do not favor the use of temozolomide as first-line therapy in patients with TCs (Baudin *et al.* 2021). Another treatment option is the mTOR inhibitor everolimus, which is now licensed to treat unresectable or metastatic progressive TCs and ACs (based on the phase III RADIANT-4 study (Yao *et al.* 2016) and subgroup analysis (Fazio *et al.* 2018)). However, similar to temozolomide (Taherifard *et al.* 2024), the safety and tolerability profile of everolimus (Faggiano *et al.* 2016) is generally considered less favorable than that of LAN (Faggiano 2024), and some suggest that its risk/benefit profile may be more appropriate for use in patients with clinically significant disease progression (Cives & Strosberg 2018). The latest European Society for Medical Oncology guidelines recommend SSA therapy rather than everolimus as first-line treatment in TC and slowly progressing SSTR-positive lung carcinoids because it is better tolerated (Baudin *et al.* 2021).

This study has several limitations, the most important of which is that the predefined sample size was not met. The earlier-than-planned transition to the open-label treatment phase reduced the period of observation during the double-blind phase and further limited treatment comparisons and the clinical interpretation of the data. However, the median PFS in SPINET is consistent with that reported in other retrospective and prospective studies of patients with TCs and ACs. In these studies, median PFS ranged from 8.5 months in patients with progressive disease at study entry to 17.4 months in patients with mixed progression status at baseline (Sullivan *et al.* 2015, Bongiovanni *et al.* 2017, Ferolla *et al.* 2017). In addition, the median PFS of both arms in SPINET is consistent with the TTP and PFS results reported in phase III trials in patients with midgut and enteropancreatic NETs (Caplin *et al.* 2021, Rinke *et al.* 2009). The conservative estimate of median PFS (10 months for LAN and 6 months for placebo) for the sample size hypothesis was based on the limited evidence (PFS and TTP) in patients with BP-NETs (Pavel *et al.* 2011, Lopez *et al.* 2014, Sullivan *et al.* 2015, Karra *et al.* 2015 , Fazio *et al.* 2016 ) and midgut NETs (Rinke *et al.* 2009) that was available at the time of study design.

As noted earlier, the most important limitation of the study is the reduced sample size, which affected data interpretation and probably explains the lack of significance between LAN and placebo for centrally assessed PFS. In addition, progressive disease was not an entry criterion for the study, which means that it was not possible to formally evaluate disease stabilization; however, the randomized nature of the trial was implemented to overcome this limitation. Another limitation is the difference in results for central and local assessment of tumor response, which may reflect variability in the assessment of metastases and differences in target lesion selection in slowly progressive NETs, as noted in the RADIANT-2 study (Pavel *et al.* 2011), and a higher rate of false positive targets in the thoracic area. In SPINET, in two patients where central and local responses differed, the numbers of baseline target lesions were different in central and local assessments. The challenges associated with assessing baseline target lesions in patients with NETs are well known (Abramson *et al.* 2015), hence the robust central assessment process adopted in SPINET (two readers, with adjudication in the event of differences in opinion). Although some differences between local and central assessment were observed during the study, it was only in a single patient that this difference had a significant impact on the tumor response. This patient had two measurable but small target lesions at baseline (16 mm and 18 mm on central measurement and 19 mm and 18 mm on local assessment); one of these lesions became non-measurable on central assessment only. A further limitation includes minor imbalances in baseline characteristics between treatment arms; however, the final clinical relevance of these imbalances is unpredictable. It should also be noted that data on the median time from the onset of metastatic disease to the start of treatment are not available.

Strengths of the study include that it enrolled an international population and was a dedicated, prospective evaluation of early SSA therapy for TCs and ACs, and that it represents the largest prospective study to date of early SSA use in this setting. Based on data from countries permitting the collection of race data, the racial diversity of the study population was limited because most patients were European; nevertheless, disease characteristics were generally as expected of those with TCs and ACs in clinical practice. As per regulatory requirements (Zhang *et al.* 2018), there was a robust, well-established, first-line central assessment of PFS that, nonetheless, allowed individualized care of patients to continue. Thus, the study provides clinically important data.

The difficulties faced in the SPINET study prompt some important reflections on missed opportunities to provide the best level of evidence achievable through the completion of a phase III trial, especially in rare cancers. At the time SPINET was designed, conducting a dedicated phase III study was considered feasible based on the estimated incidence (~1.5 per 100,000 (Öberg *et al.* 2012)). More importantly, it was anticipated that it would offer significant benefits to participants (including those initially treated with placebo, who could transition to LAN in the open-label treatment phase), as well as patients whose treatment would be informed by its outcomes. With hindsight, optimal changes to treatment guidelines in these circumstances might have included advocating strongly for SSA treatment via clinical trial participation when possible, as an alternative to off-label prescription. It is also the case, however, that meeting the best interests of patients and increasing recruitment into the study would have been possible with increased awareness among thoracic surgeons and pneumologists, as well as improved standardization in the way in which TCs and ACs are characterized in expert NET centers and in the use of prognostic factors to guide treatment decisions.

In conclusion, despite lower-than-target enrollment, SPINET is the largest prospective study to date of SSA therapy in SSTR-positive TCs and ACs. The study provides clinically important data about the activity and tolerability profile of LAN 120 mg every 28 days in unresectable and/or metastatic BP-NETs. The results of SPINET thus provide much-needed data to support the clinical use of SSAs in BP-NETs, mainly TCs, as recommended by European and US guidelines.

## Supplementary Materials

Supplementary Material

## Declaration of interest

SS: honoraria – Advanced Accelerator Applications-Novartis, Ipsen, institutional grant funding – Advanced Accelerator Applications-Novartis. DF: research grant – Advanced Accelerator Applications-Novartis, Camurus, Ipsen, and Pfizer; advisory board – Advanced Accelerator Applications-Novartis, Bristol Myers Squibb, Camurus, Ipsen, Pfizer, and Sandoz; invited speaker – Advanced Accelerator Applications-Novartis, Ipsen, and Pfizer. EW: nothing to disclose. JC: scientific consultancy role (speaker and advisory roles) – Advanz, Amgen, Bayer, Esteve, Hutchinson Pharma, Ipsen, ITM, Eisai, Exelixis, Lilly, Merck Serono, Novartis, Pfizer, Roche, Sanofi; research support – AstraZeneca, Advanced Accelerator Applications – Novartis, Amgen, Bayer, Eisai, and Pfizer. WB: nothing to disclose. DR: advisory board – Advanced Accelerator Applications-Novartis; research funding – Ipsen, Merck, Novartis. MC: honoraria for speaker bureau and advisory boards – Advanced Accelerator Applications, Chiasma, Ipsen, Novartis, and Pfizer. ED: adviser for Ipsen, Novartis, and Roche. MR: honoraria – Eisai, Eli Lilly, Gilead, Ipsen, Novartis, and Roche. DH: advisory board – Advanz Pharma, Ipsen, and Novartis; invited speaker – Ipsen. EB: research grant – Advanced Accelerator Applications-Novartis, HRA, Pfizer, and Novartis; advisory board – Advanced Accelerator Applications-Novartis, HRA, Hutchison Pharma, and Novartis; principal investigator – Ipsen and Novartis; project lead – Ipsen. AH: employee and shareholder – Ipsen. XMTT: employee – Ipsen.

## Funding

This work was supported by Ipsenhttp://dx.doi.org/10.13039/501100014382.

## Data sharing

Anonymized patient-level trial data that underlie the results reported in this publication may be made available to researchers who provide a research proposal. Data from eligible studies are available 6 months after the studied medicine and indication have been approved in the USA and EU or after the primary manuscript describing the results has been accepted for publication, whichever is later. Additional study documentation, including the clinical study report, study protocol with any amendments, annotated case report form, statistical analysis plan, and dataset specifications, may also be made available. Patient-level data will be anonymized, and study documents will be redacted to protect the privacy of trial participants. Any requests should be submitted to www.vivli.org for assessment by an independent scientific review board. Further details on Ipsen’s sharing criteria, eligible studies, and process for sharing are available here (https://vivli.org/members/ourmembers/).

## Author contribution statement

Conception or design of the work: EB, XMTT, AH, DH; acquisition, analysis, or interpretation of data: all authors; drafting the manuscript or revising it critically for important intellectual content: all authors; final approval of the version to be published: all authors.

## References

[bib1] Abramson RG Mcghee CR Lakomkin N & Arteaga CL 2015 Pitfalls in RECIST data extraction for clinical trials: beyond the basics. Academic Radiology 22 779–786. (10.1016/j.acra.2015.01.015) 25794800 PMC4429002

[bib2] Baudin E Caplin M Garcia-Carbonero R Fazio N Ferolla P Filosso PL Frilling A De Herder WW Hörsch D Knigge U, *et al.* 2021 Lung and thymic carcinoids: ESMO Clinical Practice Guidelines for diagnosis, treatment and follow-up. Annals of Oncology 32 439–451. (10.1016/j.annonc.2021.01.003) 33482246

[bib3] Bianchi A De Marinis L Fusco A Lugli F Tartaglione L Milardi D Mormando M Lassandro AP Paragliola R Rota CA, *et al.* 2011 The treatment of neuroendocrine tumors with long-acting somatostatin analogs: a single center experience with lanreotide autogel. Journal of Endocrinological Investigation 34 692–697. (10.3275/8058) 22067307

[bib4] Bongiovanni A Recine F Riva N Foca F Liverani C Mercatali L Nicolini S Pieri F Amadori D & Ibrahim T 2017 Outcome analysis of first-line somatostatin analog treatment in metastatic pulmonary neuroendocrine tumors and prognostic significance of (18)FDG-PET/CT. Clinical Lung Cancer 18 415–420. (10.1016/j.cllc.2016.11.004) 27956089

[bib5] Caplin ME Baudin E Ferolla P Filosso P Garcia-Yuste M Lim E Oberg K Pelosi G Perren A Rossi RE, *et al.* 2015 Pulmonary neuroendocrine (carcinoid) tumors: European Neuroendocrine Tumor Society expert consensus and recommendations for best practice for typical and atypical pulmonary carcinoids. Annals of Oncology 26 1604–1620. (10.1093/annonc/mdv041) 25646366

[bib6] Caplin ME Pavel M Ćwikła JB Phan AT Raderer M Sedláčková E Cadiot G Wolin EM Capdevila J Wall L, *et al.* 2014 Lanreotide in metastatic enteropancreatic neuroendocrine tumors. New England Journal of Medicine 371 224–233. (10.1056/NEJMoa1316158) 25014687

[bib7] Caplin ME Pavel M Phan AT Ćwikła JB Sedláčková E Thanh XT Wolin EM Ruszniewski P & CLARINET Investigators 2021 Lanreotide autogel/depot in advanced enteropancreatic neuroendocrine tumours: final results of the CLARINET open-label extension study. Endocrine 71 502–513. (10.1007/s12020-020-02475-2) 33052555 PMC7881960

[bib8] Cives M & Strosberg JR 2018 Gastroenteropancreatic neuroendocrine tumors. CA 68 471–487. (10.3322/caac.21493) 30295930

[bib9] Del Olmo-Garcia MI Prado-Wohlwend S Andres A Soriano JM Bello P & Merino-Torres JF 2021 Somatostatin and somatostatin receptors: from signaling to clinical applications in neuroendocrine neoplasms. Biomedicines 9 1810. (10.3390/biomedicines9121810) 34944626 PMC8699000

[bib10] Dromain C Loaiza-Bonilla A Mirakhur B Beveridge TJR & Fojo AT 2021 Novel tumor growth rate analysis in the randomized CLARINET study establishes the efficacy of lanreotide depot/autogel 120 mg with prolonged administration in indolent neuroendocrine tumors. Oncologist 26 e632–e638. (10.1002/onco.13669) 33393112 PMC8018300

[bib11] Eisenhauer EA Therasse P Bogaerts J Schwartz LH Sargent D Ford R Dancey J Arbuck S Gwyther S Mooney M, *et al.* 2009 New response evaluation criteria in solid tumours: revised RECIST guideline (version 1.1). European Journal of Cancer 45 228–247. (10.1016/j.ejca.2008.10.026) 19097774

[bib12] Faggiano A 2024 Long-acting somatostatin analogs and well differentiated neuroendocrine tumors: a 20-year-old story. Journal of Endocrinological Investigation 47 35–46. (10.1007/s40618-023-02170-9) 37581846 PMC10776682

[bib13] Faggiano A Malandrino P Modica R Agrimi D Aversano M Bassi V Giordano EA Guarnotta V Logoluso FA Messina E, *et al.* 2016 Efficacy and safety of everolimus in extrapancreatic neuroendocrine tumor: a comprehensive review of literature. Oncologist 21 875–886. (10.1634/theoncologist.2015-0420) 27053503 PMC4943387

[bib14] Fazio N Buzzoni R Delle Fave G Tesselaar M Wolin E Van Cutsem E Tomassetti P Strosberg J Voi M Pacaud LB, *et al.* 2016 Efficacy and safety of everolimus in advanced, progressive, nonfunctional neuroendocrine tumors (NET) of the lung: a subgroup analysis of the phase 3 RADIANT-4 study. Presented at NANETS 2016, abstract C04. 9th Annual Multidisciplinary NET Symposium Jackson, WY, USA. Albany, NY, USA: NANETS.(available at: https://nanets.net/abstracts-archive/2016/435-c4-efficacy-and-safety-of-everolimus-in-advanced-progressive-nonfunctional-neuroendocrine-tumors-net-of-the-lung-a-subgroup-analysis-of-the-phase-3-radiant-4-study/file)

[bib15] Fazio N Buzzoni R Delle Fave G Tesselaar ME Wolin E Van Cutsem E Tomassetti P Strosberg J Voi M Bubuteishvili-Pacaud L, *et al.* 2018 Everolimus in advanced, progressive, well-differentiated, non-functional neuroendocrine tumors: RADIANT-4 lung subgroup analysis. Cancer Science 109 174–181. (10.1111/cas.13427) 29055056 PMC5765303

[bib16] Ferolla P Berruti A Spada F Brizzi MP Ibrahim T Marconcini R Giuffrida D Amoroso V La Salvia A Vaccaro V, *et al.* 2023 Efficacy and safety of lanreotide autogel and temozolomide combination therapy in progressive thoracic neuroendocrine tumors (carcinoid): results from the phase 2 ATLANT study. Neuroendocrinology 113 332–342. (10.1159/000526811) 36044870

[bib17] Ferolla P Brizzi MP Meyer T Mansoor W Mazieres J Do Cao C Léna H Berruti A Damiano V Buikhuisen W, *et al.* 2017 Efficacy and safety of long-acting pasireotide or everolimus alone or in combination in patients with advanced carcinoids of the lung and thymus (LUNA): an open-label, multicentre, randomised, phase 2 trial. Lancet: Oncology 18 1652–1664. (10.1016/S1470-2045(1730681-2) 29074099

[bib18] Filosso PL Falcoz PE Solidoro P Pellicano D Passani S Guerrera F Ruffini E & ESTS Lung Neuroendocrine Working-Group Participating Centers 2018 The European Society of Thoracic Surgeons (ESTS) lung neuroendocrine tumors (NETs) database. Journal of Thoracic Disease 10(Supplement 29) S3528–S3532. (10.21037/jtd.2018.04.104) 30510790 PMC6230831

[bib19] Godara A Siddiqui NS Byrne MM & Saif MW 2019 The safety of lanreotide for neuroendocrine tumor. Expert Opinion on Drug Safety 18 1–10. (10.1080/14740338.2019.1559294) 30582380

[bib20] Ito T Fujimori N Honma Y Kudo A Hijioka S Katsushima S Kimura Y Fukutomi A Hisamatsu S Nakajima A, *et al.* 2021 Long-term safety and efficacy of lanreotide autogel in Japanese patients with neuroendocrine tumors: final results of a phase II open-label extension study. Asia-Pacific Journal of Clinical Oncology 17 e153–e161. (10.1111/ajco.13371) 32757459 PMC8596629

[bib21] Karra E Polycarpou A Tanaskovic N Garcia-Hernandez J Mullan M Caplin M & Toumpanakis C 2015 Somatostatin analogues in bronchial neuroendocrine tumors: symptom control and anti-proliferative role. Neuroendocrinology 102 77–168. (10.1159/000431385)

[bib22] Kulke MH Shah MH Benson AB 3rd Bergsland E Berlin JD Blaszkowsky LS Emerson L Engstrom PF Fanta P Giordano T, *et al.* 2015 Neuroendocrine tumors, version 1.2015. Journal of the National Comprehensive Cancer Network 13 78–108. 25583772 10.6004/jnccn.2015.0011

[bib23] Kunz PL Reidy-Lagunes D Anthony LB Bertino EM Brendtro K Chan JA Chen H Jensen RT Kim MK Klimstra DS, *et al.* 2013 Consensus guidelines for the management and treatment of neuroendocrine tumors. Pancreas 42 557–577. (10.1097/MPA.0b013e31828e34a4) 23591432 PMC4304762

[bib24] Lopez C Jiménez-Fonseca P Capdevila J Alonso T Custodio A Crespo G Aller J Madrid C Gomez-Roman J& Grande E 2014 SSA therapy for patients with bronchial carcinoids (BC) in the community setting. Neuroendocrinology 99 219–310. (10.1159/000367792)

[bib25] Martín-Richard M Massutí B Pineda E Alonso V Marmol M Castellano D Fonseca E Galán A Llanos M Sala MA, *et al.* 2013 Antiproliferative effects of lanreotide autogel in patients with progressive, well-differentiated neuroendocrine tumours: a Spanish, multicentre, open-label, single arm phase II study. BMC Cancer 13 427. (10.1186/1471-2407-13-427) 24053191 PMC3853091

[bib26] Michael M Garcia-Carbonero R Weber MM Lombard-Bohas C Toumpanakis C & Hicks RJ 2017 The antiproliferative role of lanreotide in controlling growth of neuroendocrine tumors: a systematic review. Oncologist 22 272–285. (10.1634/theoncologist.2016-0305) 28220021 PMC5344642

[bib27] Öberg K Hellman P Ferolla P Papotti M & ESMO Guidelines Working Group 2012 Neuroendocrine bronchial and thymic tumors: ESMO Clinical Practice Guidelines for diagnosis, treatment and follow-up. Annals of Oncology 23(Supplement 7) vii120–vii123. (10.1093/annonc/mds267) 22997444

[bib28] Pavel ME Hainsworth JD Baudin E Peeters M Hörsch D Winkler RE Klimovsky J Lebwohl D Jehl V Wolin EM, *et al.* 2011 Everolimus plus octreotide long-acting repeatable for the treatment of advanced neuroendocrine tumours associated with carcinoid syndrome (RADIANT-2): a randomised, placebo-controlled, phase 3 study. Lancet 378 2005–2012. (10.1016/S0140-6736(1161742-X) 22119496

[bib29] Rinke A Maintz C Müller L Weber MM Lahner H Pavel M Saeger W Houchard A Ungewiss H & Petersenn S 2021 Multicenter, observational study of lanreotide autogel for the treatment of patients with neuroendocrine tumors in routine clinical practice in Germany and Austria. Experimental and Clinical Endocrinology and Diabetes 129 500–509. (10.1055/a-1342-2755) 34293802 PMC8298132

[bib30] Rinke A Müller HH Schade-Brittinger C Klose KJ Barth P Wied M Mayer C Aminossadati B Pape UF Bläker M, *et al.* 2009 Placebo-controlled, double-blind, prospective, randomized study on the effect of octreotide LAR in the control of tumor growth in patients with metastatic neuroendocrine midgut tumors: a report from the PROMID Study Group. Journal of Clinical Oncology 27 4656–4663. (10.1200/JCO.2009.22.8510) 19704057

[bib31] Robelin P Hadoux J Forestier J Planchard D Hervieu V Berdelou A Scoazec JY Valette PJ Leboulleux S Ducreux M, *et al.* 2019 Characterization, prognosis, and treatment of patients with metastatic lung carcinoid tumors. Journal of Thoracic Oncology 14 993–1002. (10.1016/j.jtho.2019.02.002) 30771520

[bib32] Sullivan I Baudin E Guigay J Scoazec JY Leboulleux S Berdelou A Ducreux M Malka D Caramella C Besse B, *et al.* 2015 Tumor control of advanced pulmonary carcinoid tumors with somatostatin analogs: experience at Gustave Roussy. European Journal of Cancer 51 S622–S623. (10.1016/S0959-8049(1631717-8)

[bib33] Taherifard E Bakhtiar M Mahnoor M Ahmed R Cavalcante L Zhang J & Saeed A 2024 Efficacy and safety of temozolomide-based regimens in advanced pancreatic neuroendocrine tumors: a systematic review and meta-analysis. BMC Cancer 24 192. (10.1186/s12885-024-11926-2) 38347461 PMC10860315

[bib34] Vesterinen T Leijon H Mustonen H Remes S Knuuttila A Salmenkivi K Vainio P Arola J & Haglund C 2019 Somatostatin receptor expression is associated with metastasis and patient outcome in pulmonary carcinoid tumors. Journal of Clinical Endocrinology and Metabolism 104 2083–2093. (10.1210/jc.2018-01931) 30657933

[bib35] Yao JC Fazio N Singh S Buzzoni R Carnaghi C Wolin E Tomasek J Raderer M Lahner H Voi M, *et al.* 2016 Everolimus for the treatment of advanced, non-functional neuroendocrine tumours of the lung or gastrointestinal tract (RADIANT-4): a randomised, placebo-controlled, phase 3 study. Lancet 387 968–977. (10.1016/S0140-6736(1500817-X) 26703889 PMC6063317

[bib36] Zhang J Zhang Y Tang S Jiang L He Q Hamblin LT He J Xu Z Wu J Chen Y, *et al.* 2018 Systematic bias between blinded independent central review and local assessment: literature review and analyses of 76 phase III randomised controlled trials in 45 688 patients with advanced solid tumour. BMJ Open 8 e017240. (10.1136/bmjopen-2017-017240) PMC6144327 30206071

