# Supplementary material for: Treatment of advanced BP-NETS with lanreotide autogel/depot vs placebo: the phase III SPINET study
Source: Endocr Relat Cancer. 2024 Jul 22;31(9):e230337. doi: 10.1530/ERC-23-0337 (PMC11301421; doi:10.1530/ERC-23-0337)
Supplement: Supplementary Material [file supplementary_material.pdf]

Supplementary material

Randomization and blinding

- The randomization schedule was created from computer-generated randomization lists by a statistician from the sponsor company who was independent from the SPINET study.
- After eligibility was confirmed by study investigators, patients were assigned a randomization number in sequential order in blocks of six within each stratum (tumor subtype, typical carcinoid vs. atypical carcinoid; prior vs. no prior chemotherapy [cytotoxic, molecular targeted therapy, or interferon-alpha]) by the investigator using the interactive web response system (IWRS). The investigator also used the IWRS to assign treatment numbers for study medication during both the double-blind and open-label treatment phases.
- During the double-blind phase, patients and all study personnel, including those involved in reading the magnetic resonance imaging or computed tomography scans, were blinded to treatment allocation.
    - The exceptions were a trained nurse who was appointed by the investigator to administer treatment (because lanreotide autogel/depot [LAN] and placebo were not similar in color and appearance) and a member of the sponsor’s clinical operations team, who was responsible for managing any emergencies.
- In the event of an issue related to patient safety, investigators were able to break the treatment code (unblind study treatment) via the IWRS.

Biomarker assessment

- Secondary endpoints of the study included the change from baseline in serum chromogranin (CgA) levels, the proportion of patients with a ≥30% decrease in CgA at week 8 in patients with elevated baseline levels (≥2 × upper limit of normal [ULN]), and the change from baseline in urinary 5-hydroxyindoleacetic acid (5-HIAA) levels in patients with elevated baseline levels (≥2 × ULN).

RECIST responses during the double-blind phase: central compared with local assessment

During the double-blind phase, eight patients in the LAN group had better responses for central than for local assessment: three had stable disease on central assessment compared with progressive disease (PD) on local assessment, three had a partial response (PR) compared with stable disease, and two had a complete response compared with stable disease (n=1) or PR (n=1) (see Table (a) below). One patient in

the LAN group had a worse response on central assessment (PD versus stable disease on local assessment). In the placebo group, one patient had a better response for central (stable disease) compared with local assessment (PD) and two patients had a worse response (PD vs. stable disease and stable disease vs. PR) (see Table (b) below).

(a) LAN group

	Centrally assessed					
Locally assessed		NE	PD	Stable disease	PR	CR
	PD	1	3	3		
	Stable disease		1	35	3	1
	PR				2	1

(b) Placebo group

	Centrally assessed					
Locally assessed		NE	PD	Stable disease	PR	CR
	PD		1	1		
	Stable disease		1	21		
	PR	1		1		

Data from four patients were not in adequation between central and local review, i.e., they were classified as having PR or CR on central review, but SD on local review. Two of these four patients had a sum of diameters (SOD) for target lesions more than 50 mm. In the first patient, the SOD was 116 mm on central assessment and 123 mm on local assessment; in the second patient, the SOD was 55 mm and 63 mm, respectively.

For two of the patients not in adequation (Patients 1 and 3 in Table (c) below), the number of target lesions at baseline differed between local and central assessment, and in one further patient (Patient 2 in Table (c) below), the tumor became non-measurable on central but not local assessment.

(c) SOD for target lesions for patients not in adequation for central and local assessment

Patient no.	Time point	Target lesion length (mm)	Central	Local
1	Baseline	T1	20	12
		T2	16	10
		T3	10	NA
		SOD	46	22
	Week 12	T1	7	10
		T2	10	10
		T3	7	NA
		SOD	24	20
2	Baseline	T1	16	19
		T2	18	18
		SOD	34	37
	Week 12	T1	10	15
		T2	18	12
		SOD	28	27
	Week 24	T1	0	17
		T2	20	13
		SOD	20	30
3	Baseline	T1	29	23
		T2	26	22
		T3	NA	18
		SOD	55	63
	Week 12	T1	27	28
		T2	26	28
		T3	NA	18
		SOD	53	74
	Week 24	T1	28	26
		T2	26	25
		T3	NA	22
		SOD	54	73
	Week 36	T1	25	25
		T2	33	25
		T3	NA	24
		SOD	58	74
	Week 48	T1	18	25
		T2	0	25
		T3	NA	25
		SOD	18	75

Gallbladder echography

- One patient in each treatment group had a history of cholelithiasis.
- Seven patients in the LAN group and two in the placebo group underwent gallbladder echography at baseline; one had lithiasis (in LAN group) and two had sludge (one in each group).
    - The patient in the placebo group with baseline sludge developed lithiasis by week 24.
- None of the other patients who underwent post-baseline gallbladder echography (n=2, both in the LAN group) developed new sludge or new lithiasis during the double-blind or open-label treatment phases.
    - In the patient in the LAN group with lithiasis and sludge at baseline, both were still present at week 8. The patient had gallbladder surgery shortly after.

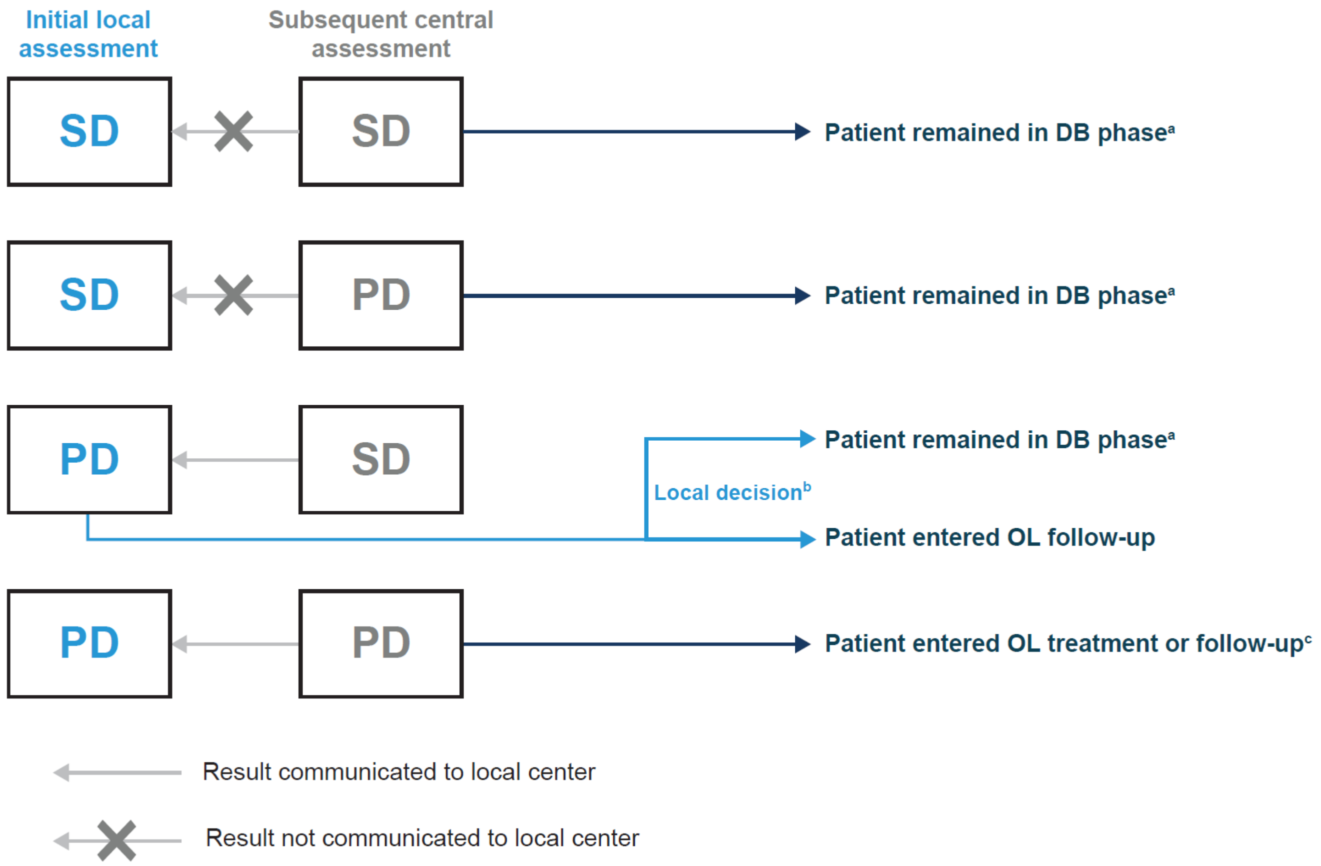

Supplementary Figure S1. Radiology review procedures

Central assessment = two reviewers and an adjudicator if necessary.

In the event of a difference in assessment between central reviewers, a third, independent, blinded adjudicator reassessed the scans. [a]After recruitment was stopped (protocol amendment), all patients could switch into the open-label treatment phase; [b]If locally assessed disease progression was not confirmed centrally, the local decision prevailed; [c]In the event of PD during the double-blind phase, patients in the LAN group entered the open-label follow-up, whereas patients in the placebo group had the option to enter the open-label treatment phase; in the event of PD

during open-label treatment, patients entered the open-label follow-up phase.

DB, double-blind; LAN, lanreotide autogel/depot; OL, open-label; PD, progressive disease; SD, stable disease

Supplementary Figure S2. Changes in EORTC QLQ-C30 scores during the DB phase (ITT population)

(a) Global health status score

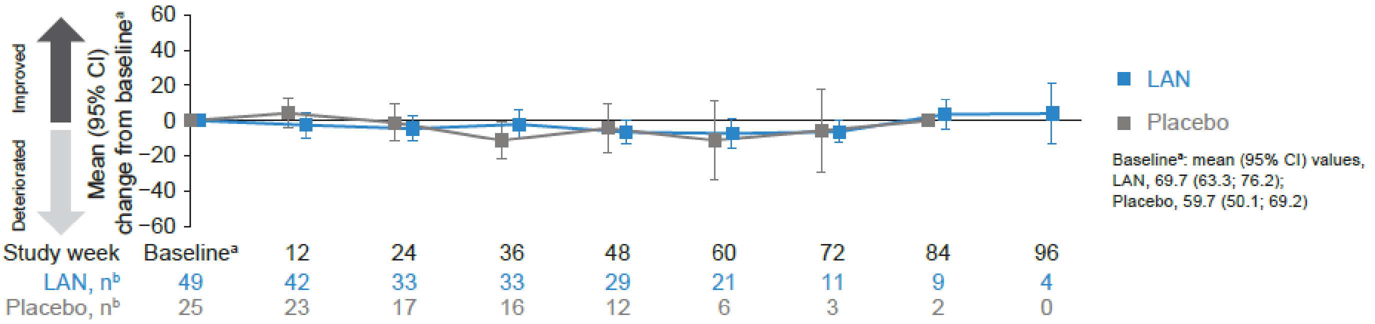

(b) Diarrhea

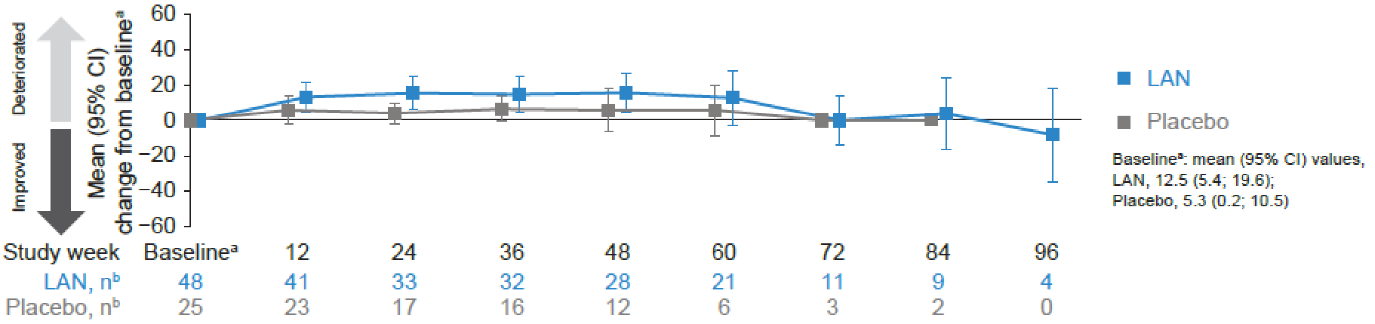

(c) Fatigue

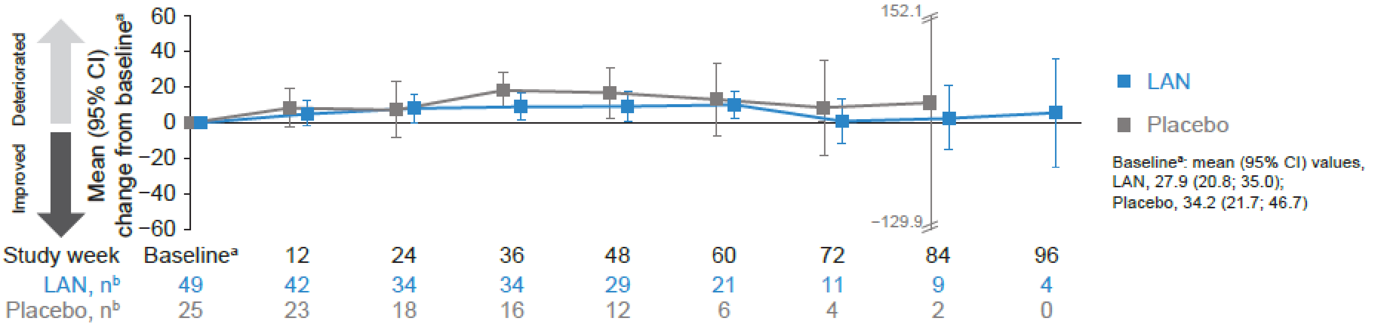

(d) Pain

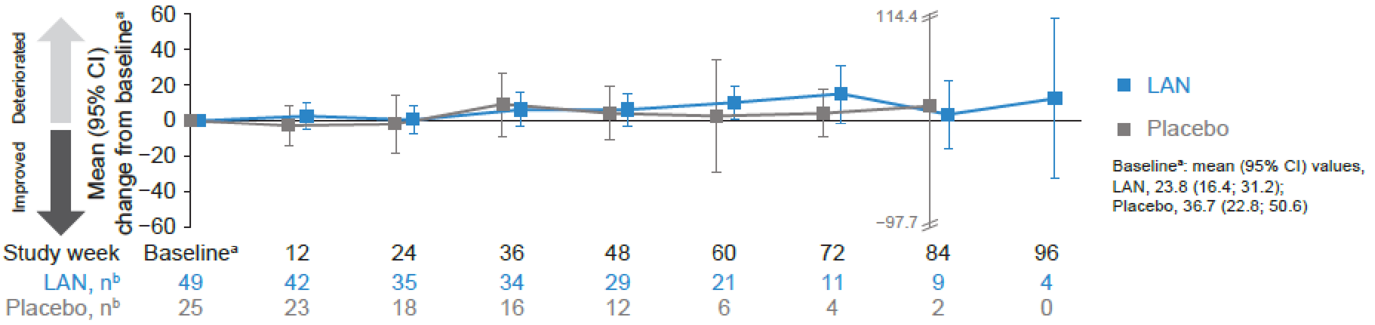

[a]Last non-missing measurement before first dose (DB baseline). [b]Patients with baseline data/available change from baseline data. Absent 95% CIs were noncalculable.

CI, confidence interval; DB, double-blind; EORTC, European Organisation for Research and Treatment of Cancer; ITT, intention-to-treat; LAN, lanreotide autogel/depot; QLQ-C30, Quality of Life Questionnaire Core 30

Supplementary Figure S3. EORTC QLQ-C30 scores during the open-label treatment phase (open-label ITT population)

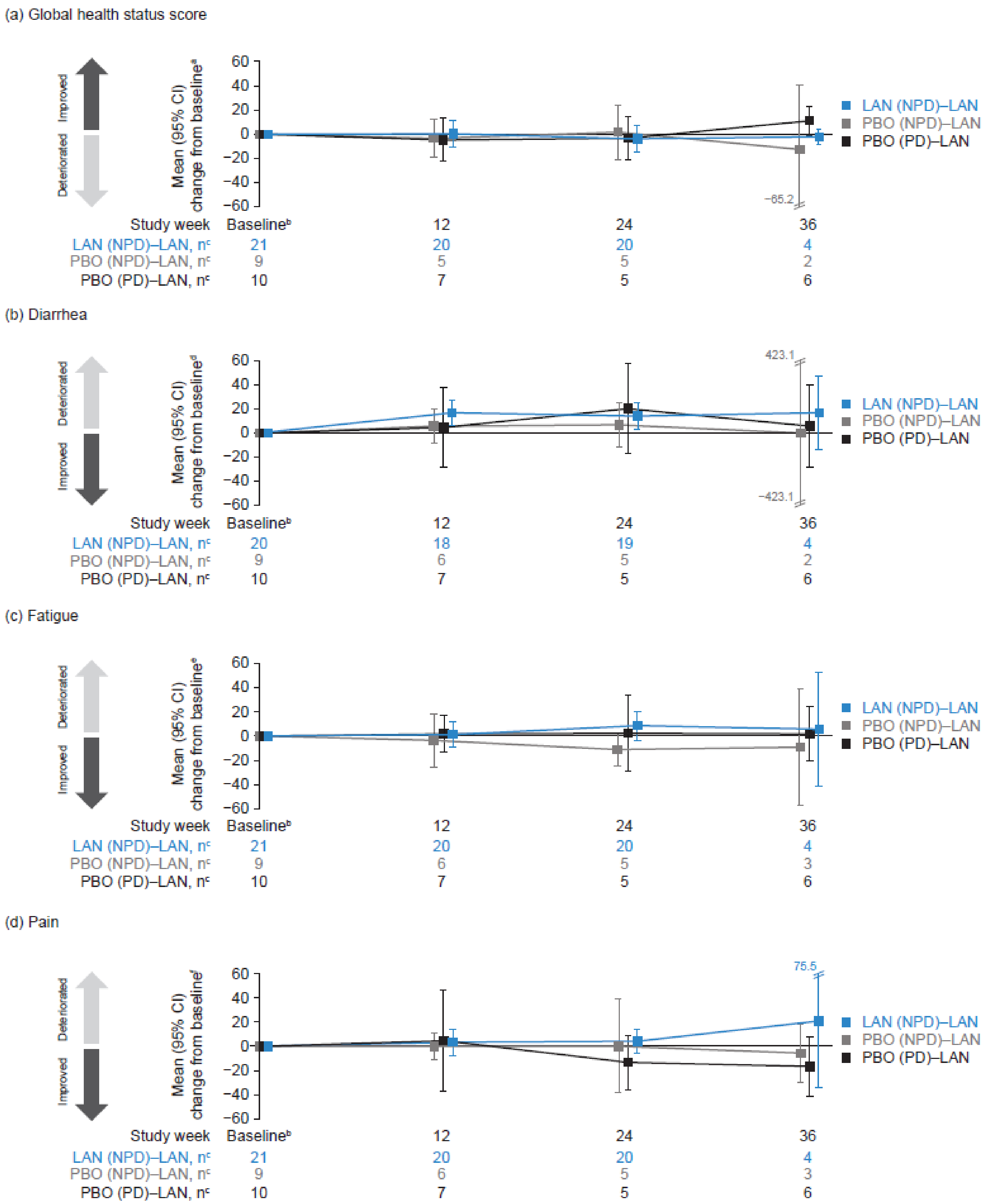

[a]Mean (95% CI) baseline[b] values were 73.0 (64.0; 82.1) in the LAN (NPD)–LAN group, 61.1 (46.4; 75.8) in the PBO (NPD)–LAN group and 55.8 (36.1; 75.5) in the PBO (PD)–LAN group. [b]Last non-missing measurement before first LAN dose (DB baseline or open-label baseline). [c]Patients with baseline data/available change from baseline data. [d]Mean (95% CI) baseline[b] values were 10.0 (-1.4; 21.4) in the LAN (NPD)–LAN group, 3.7 (-4.8; 12.2) in the PBO (NPD)–LAN group and 16.7 (-6.5; 39.8) in the PBO (PD)–LAN group; [e]Mean (95% CI) baseline[b] values were 24.8 (15.7; 34.0) in the LAN (NPD)–LAN group, 28.4 (14.1; 42.6) in the PBO (NPD)–LAN group and 51.1 (31.2; 71.0) in the PBO (PD)–LAN group. [f]Mean (95% CI) baseline[b] values were 17.5 (8.0; 26.9) in the LAN (NPD)–LAN group, 24.1 (2.7; 45.4) in the PBO (NPD)–LAN group and 40.0 (18.1; 61.9) in the PBO (PD)–LAN group. Data were also collected at weeks 48, 60, 72, and 84 for the PBO (PD)–LAN group, but the data are not shown because the patient numbers were very low (6, 4, 3, and 1, respectively).

Absent 95% CIs were noncalculable.

CI, confidence interval; DB, double-blind; EORTC, European Organisation for Research and Treatment of Cancer; ITT, intention-to-treat; LAN, lanreotide autogel/depot; NPD, no progressive disease; PBO, placebo; PD, progressive disease; QLQ C30, Quality of Life Questionnaire Core 30.

Supplementary Figure S4. Changes in serum CgA levels during the DB phase

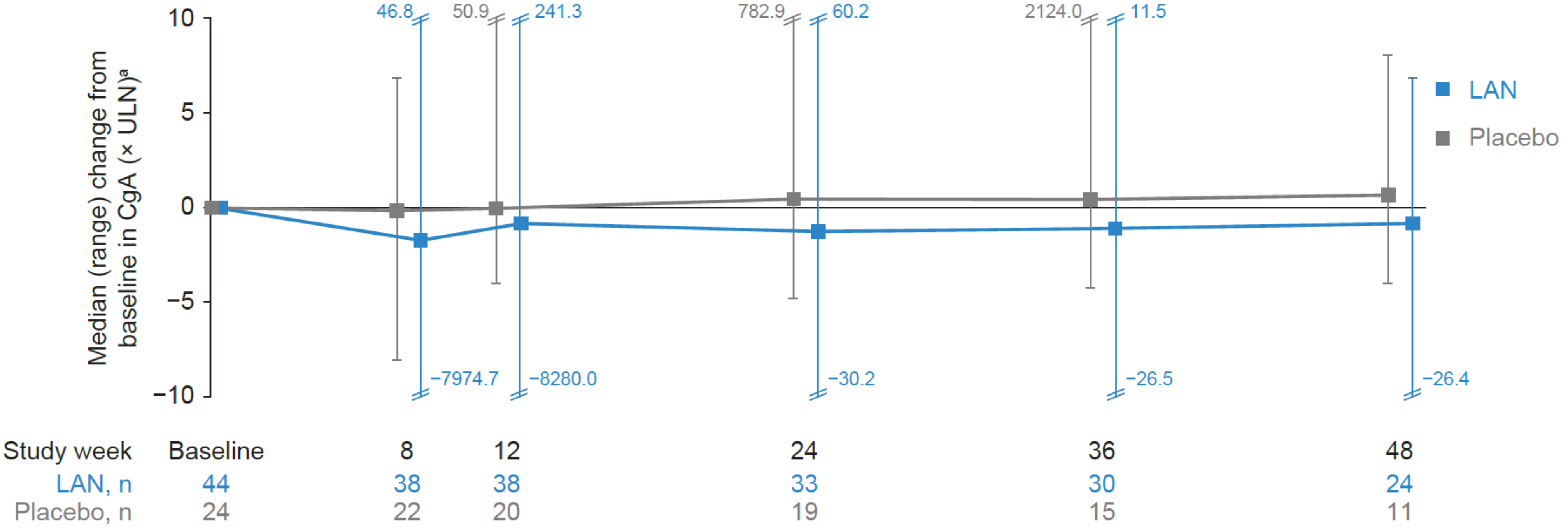

[a]Median (range) baseline values (× ULN) were 5.15 (0.3–14,442.1) in the LAN group and 2.12 (0.5–585.5) in the placebo group.

CgA, chromogranin A; DB, double-blind; LAN, lanreotide autogel/depot; ULN, upper limit of normal.

Supplementary Figure S5. Changes in urinary 5-HIAA levels during the DB phase in patients with elevated baseline levels (≥2 × ULN)

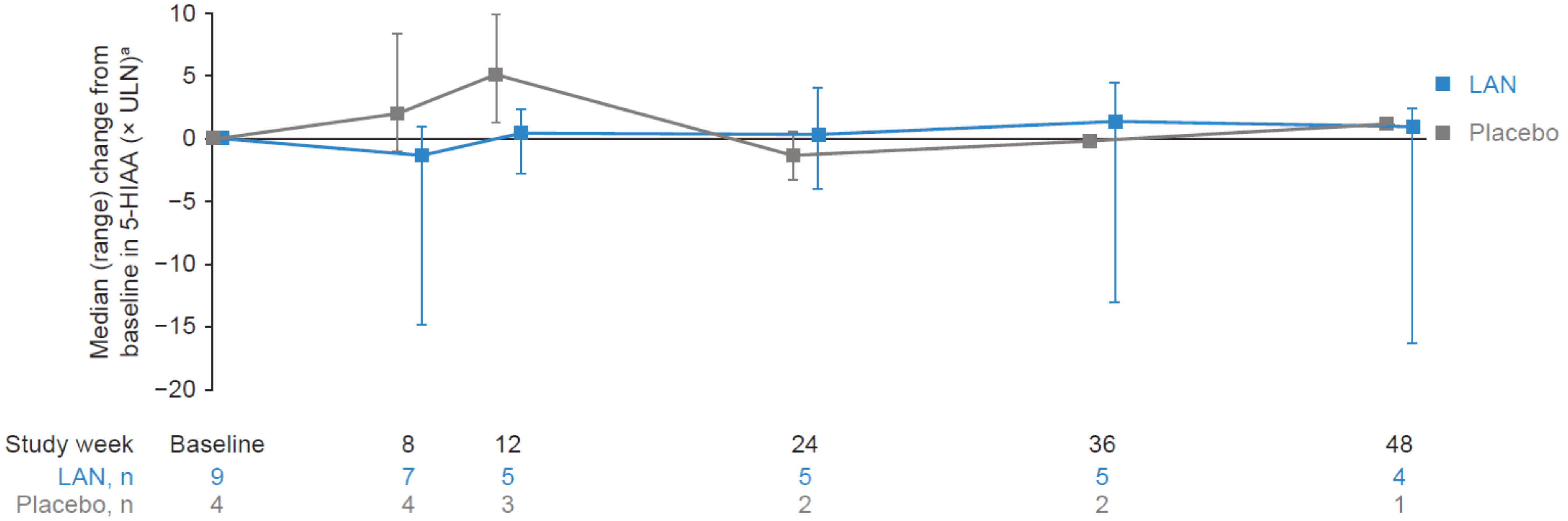

[a]Median (range) baseline values (× ULN) were 4.47 (2.1–16.9) in the LAN group and 6.33 (2.4–29.1) in the placebo group

5-HIAA, 5-hydroxyindoleacetic acid; DB, double-blind; LAN, lanreotide autogel/depot; ULN, upper limit of normal

Supplementary Table S1. Inclusion and exclusion criteria

Inclusion criteria

- Patients who provided written informed consent prior to any study-related procedures
- Age ≥18 years
- Metastatic and/or unresectable, pathologically confirmed, well-differentiated typical or atypical NETs of the lung
- Histological evidence of well-differentiated NETs of the lung (typical and atypical according to the WHO criteria evaluated locally)
- Mitotic index <2 mitoses/2 mm^2 (TC) or ≤10 mitoses/2 mm^2 (TC) and/or foci of necrosis (AC)
- At least one measurable lesion on MRI or CT scan (RECIST v1.1) (Eisenhauer et al., 2009)
- Positive SSTR imaging (Krenning scale score ≥2 on Octreoscan® or uptake greater than liver background on gallium–positron emission tomography scan)
- ECOG PS scale score of 0 or 1 (ECOG-ACRIN, 2020)
- Female patients of childbearing potential had to have a negative urine or serum pregnancy test in the 72 hours before randomization; if the urine test was positive or could not be confirmed as negative, a serum pregnancy test was required
- Female patients who were at risk of becoming pregnant had to agree to use an effective method of contraception such as double barrier contraception, an injectable, combined oral contraceptive, or an intra-uterine device. The patient had to agree to use the contraception during the whole period of the study and for 8 months after the last study treatment administration. Non-childbearing potential was defined as being postmenopausal for ≥1 year, or permanently sterilized at least 3 months before study entry
- Male patients had to agree that, if their partner was at risk of becoming pregnant, they would use an effective method of contraception (see above). The patient had to agree to use the contraception during the whole period of the study and for 8 months after the last study treatment administration
- Signed health insurance portability and accountability authorization when required
- Patients had to be willing and able to comply with study restrictions and to remain at the clinic for the required duration during the study period, and willing to return to the study site for the follow-up

evaluation as specified in the protocol

Exclusion criteria

- Poorly differentiated or high-grade carcinoma, or NETs not of lung origin
- Multiple endocrine neoplasia type 1
- Previous SSA treatment, except if treatment was for <15 days with a short-acting SSA or patients had received one dose of a long-acting SSA >6 weeks before randomization
- Peptide receptor radionuclide therapy at any time before randomization
- Chemotherapy (cytotoxic, molecular targeted therapy or interferon-alpha) for TCs/ACs in the 4 weeks before randomization or >2 lines of chemotherapy for TCs/ACs
- Surgery or local therapy (e.g., chemo-embolization, bland, or radio-embolization) in the 6 weeks before randomization. Patients who had previously received local therapy had to have documented growth of measurable disease within the embolization field prior to the study.
- Functional disease requiring SSA treatment for symptom management
- Patients with known ectopic production of adrenocorticotropic hormone and those with other hormone-secreting conditions were only allowed to take part if their symptoms were adequately controlled without SSAs
- Concomitant use of a growth hormone antagonist, cyclosporine, or bromocriptine
- Inadequate bone marrow function, as per the investigator’s judgment
- Severe renal insufficiency (calculated creatinine clearance <30 mL/min)
- Total bilirubin, lipase, or amylase >2 × ULN; AST, ALT or alkaline phosphatase >5 × ULN
- Serum albumin <3.0 g/dL unless prothrombin time within normal range
- Known hypersensitivity to the study treatment
- Present cholecystitis
- Uncontrolled congestive heart failure
- Glycosylated hemoglobin (hemoglobin A_{1c}) >8.5%
- Abnormal findings, any other medical condition(s), or laboratory findings that, in the opinion of the Investigator, would compromise the patient’s safety or the outcome of the study
- Other known co-existing malignancies except non-melanoma skin cancer and carcinoma *in situ* of

the uterine cervix, unless definitively treated and proven no evidence of recurrence for 5 years

- Pregnant or lactating women or those of childbearing potential age and not practicing a medically acceptable method for birth control
- Participation in any therapeutic clinical study/receipt of any investigational agent within 30 days of randomization
- Clinically significant cardiac arrhythmia, bradycardia, or tachycardia that would compromise patient safety or the outcome of the study
- Uncontrolled hypothyroidism

AC, atypical carcinoid; ALT, alanine aminotransferase; AST, aspartate aminotransferase; CT, computed tomography; ECOG, Eastern Cooperative Oncology Group; MRI, magnetic resonance imaging; NET, neuroendocrine tumor; PS, performance status; RECIST, Response Evaluation Criteria in Solid Tumors; SSA, somatostatin analog; SSTR, somatostatin receptor; TC, typical carcinoid; ULN, upper limit of normal; WHO, World Health Organization

Supplementary Table S2. Schedule of key assessments during the double-blind phase

			Time (weeks)												Every 12 weeks thereafter	Post-treatment or early withdrawal
	**Screening**	**Baseline[a]**	**4**	**8**	**12**	**16**	**20**	**24**	**28**	**32**	**36**	**40**	**44**	**48**		
CT or MRI[b]	X	X			X			X			X			X	X	X
EORTC QLQ-C30[c]		X			X			X			X			X	X	X
Serum CgA and urinary 5-HIAA[d]		X		X	X			X			X			X	X	X
Adverse events	Throughout the study															
Hematology and biochemistry	X	X			X			X			X			X	X	X
Vital signs	X	X		X	X			X			X			X	X[e]	X
Electrocardiogram	X							X						X	X	X
Gallbladder echography[f]	Performed if biological abnormalities and/or clinical symptoms of gallbladder inflammation present[g]															

[a]The baseline assessment was performed on day 1, prior to treatment; the screening assessments could serve as baseline if performed within 3 days (72 hours) before day 1 (randomization); [b]CT/MRI of the thorax and abdomen was performed in the 28 days before day 1 (or if they were performed within 12 months of baseline as part of an ancillary study, they were sent for analysis by central review); during the double-blind phase, tumor assessments (by RECIST v1.1) were performed every 12 weeks; at the post-treatment/early withdrawal visit, scans were conducted only if not performed within the previous 4 weeks; [c]To reduce all sources of potential bias, it was recommended that the questionnaire was completed before seeing the physician; [d]Urinary 5-HIAA was measured at baseline, and if elevated or clinically indicated, again at week 8, week 12, every 12 weeks thereafter, and at the post-treatment/early withdrawal visit; [e]Performed at screening, week 24, week 48, every 24 weeks thereafter and at the post-treatment or early withdrawal visit; [f]Patients being followed with abdominal MRI did not require gallbladder echography; [g]Gallbladder inflammatory changes were included in the study discontinuation criteria

5-HIAA, 5-hydroxyindoleacetic acid; CgA, chromogranin A; CT, computed tomography; EORTC, European Organisation for Research and Treatment of Cancer; MRI, magnetic resonance imaging; QLQ-C30, Quality of Life Questionnaire Core 30; RECIST, Response Evaluation Criteria in Solid Tumors

Supplementary Table S3: Schedule of key assessments during the open-label phase

	Open-label treatment phase baseline	Every 12 weeks	Post-treatment or early withdrawal
CT or MRI[a]	X	X	X
EORTC QLQ-C30	X	X	X
Serum CgA and urinary 5-HIAA[b]	X	X	X
Adverse events		Throughout the study	
Hematology and biochemistry	X	X	X
Vital signs	X	X	X
Electrocardiogram	X	X	X
Gallbladder echography	Performed if biological abnormalities and/or clinical symptoms of gallbladder inflammation present		

[a]CT/MRI of the thorax and abdomen were performed at baseline (if not performed within the previous 4 weeks; CT/MRI used for disease progression documentation could be used if performed in the previous 4 weeks) and every 12 weeks; at the post-treatment/early withdrawal visit, scans were conducted only if not performed in the previous 4 weeks; [b]Urinary 5-HIAA was assessed only if elevated (≥2 × ULN) at baseline of the double-blind phase or if clinically indicated

5-HIAA, 5-hydroxyindoleacetic acid; CgA, chromogranin A; CT, computed tomography; EORTC, European Organisation for Research and Treatment of Cancer; MRI, magnetic resonance imaging; QLQ-C30, Quality of Life Questionnaire Core 30; ULN, upper limit of normal

Supplementary Table S4. TNM stage at baseline (ITT population)

	**LAN (*N* = 51)**	**Placebo (*N* = 26)**
Tumor		
TX	13 (25.5)	4 (15.4)
T0	0	0
Tis	0	0
T1	13 (25.5)	7 (26.9)
T2	13 (25.5)	7 (26.9)
T3	5 (9.8)	2 (7.7)
T4	3 (5.9)	3 (11.5)
Missing	4 (7.8)	3 (11.5)
Node		
NX	12 (23.5)	3 (11.5)
N0	21 (41.2)	9 (34.6)
N1	3 (5.9)	6 (23.1)
N2	9 (17.6)	3 (11.5)
N3	2 (3.9)	2 (7.7)
Missing	4 (7.8)	3 (11.5)
Metastases		
MX	1 (2.0)	0
M0	12 (23.5)	8 (30.8)
M1 all	34 (66.7)	15 (57.7)
M1	25 (73.5)	12 (80.0)
M1a	2 (5.9)	0
M1b	7 (20.6)	3 (20.0)
Missing	4 (7.8)	3 (11.5)

ITT, intention-to-treat; LAN, lanreotide autogel/depot; Tis, tumor *in situ*; TNM, tumor, node, metastasis

Supplementary Table S5. Types of event and reasons for censoring (PFS during double-blind and open-label treatment phases in patients randomized to LAN)

	**LAN (*N* = 51)**
**Events**	
Cumulative number of patients with events,[a,b] *n* (%)	29 (58.0)
Type of events, *n* (%)	
Progression (centrally confirmed)	28 (96.6)
Death between adequate assessment visits	1 (3.4)
Death before first PD assessment	0
**Censoring**	
Cumulative number of patients with censored data,[a] *n* (%)	21 (42.0)
Reasons for censoring, *n* (%)	
No baseline tumor assessment	0
Progression (locally documented) and withdrawal	2 (9.5)
No progression	13 (61.9)
Treatment discontinuation for undocumented progression[c]	0
Treatment discontinuation for toxicity or other reasons	5 (23.8)
New anticancer treatment started	0
Death or progression after more than one missed visit	0
Prohibited medication/therapy	0
Unblinded event	1 (4.8)

[a]Based on data from 50 patients; one patient was excluded from the analysis because their data were censored at baseline (because the baseline assessment was prior to randomization, this would have yielded a negative PFS); [b]Death or centrally assessed PD; [c]Not confirmed centrally

LAN, lanreotide autogel/depot; PD, progressive disease; PFS, progression-free survival

Supplementary Table S6. PFS (locally assessed) during the double-blind phase (ITT population)

	Median (95% CI), months		HR (95% CI)[a]; *P* value
	**LAN (*N* = 51[b])**	**Placebo (*N* = 26[b])**	
Overall population	14.1 (11.1; NC)	13.6 (8.3; NC)	0.93 (0.48; 1.88); *P* = 0.837[c]
Typical carcinoids[d]	NC (13.7; NC)	13.6 (8.3; NC)	–
Atypical carcinoids[e]	11.3 (4.8; 14.1)	13.9 (2.8; 16.9)	–

Data from patients who did not die and did not have confirmed disease progression were censored on the date of the last radiological assessment at which the target lesions were evaluated by local review.

[a]The HR and 95% CI were estimated using a Cox proportional hazards model stratified for tumor subtype using the exact method for ties; [b]One patient in each group was excluded from the analysis because their data were censored at baseline (because the baseline assessment was prior to randomization this would have yielded a negative PFS); [c]Log-rank test; [d]LAN, n = 29; placebo, n = 16; [e]LAN, n = 22; placebo n = 10.

PFS in months was calculated as follows: (date of event – date of randomization)/30.4375.

CI, confidence interval; HR, hazard ratio; ITT, intention-to-treat; LAN, lanreotide autogel/depot; NC, not calculable; PFS, progression-free survival.

Supplementary Table S7. Reasons for treatment failure (ITT population)

	LAN (*N* = 51)	Placebo (*N* = 26)
**Events**	***n* = 51**	***n* = 26**
Cumulative number of patients with events, *n* (%)	36 (70.6)	21 (80.8)
Types of event, *n* (%)	*n = 36*	*n = 21*
Progression documented[a]	29 (80.6)	16 (76.2)
Treatment discontinuation for undocumented disease progression	1 (2.8)	0
Treatment discontinuation for toxicity or any other reason	1 (2.8)	2 (9.5)
Consent withdrawn	3 (8.3)	1 (4.8)
Adverse event	0	1 (4.8)
Lost to follow-up	1 (2.8)	0
Unblinded event	1 (2.8)	1 (4.8)
**Censoring**		
Cumulative number of patients with censored data, *n* (%)	*n* = 15	*n* = 5
No progression	15 (100.0)	5 (100.0)

[a] Centrally or locally assessed.

ITT, intention-to-treat; LAN, lanreotide autogel/depot.

Supplementary Table S8. Objective response rate[a] and clinical benefit rate[b] (locally assessed; ITT population)

(a) Double-blind phase

	***n* (%) [95% CI]**		**LAN vs placebo treatment difference [95% CI]**
	**LAN (*N* = 51)**	**PBO (*N* = 26)**	
Objective response rate[a]	3/50 (6.0)<br>[1.25; 16.55]	1/25 (4.0)<br>[0.10; 20.35]	2.00<br>[–22.69; 26.53]
Clinical benefit rate[b]	43/50 (86.0)<br>[73.26; 94.18]	23/25 (92.0)<br>[73.97; 99.02]	–6.00<br>[–30.34; 18.82]

(b) Open-label treatment phase

	***n* (%) [95% CI]**				**Treatment difference [95% CI]**	
	**LAN (NPD)–LAN (*N* = 21)**	**PBO (NPD)–LAN (*N* = 9)**	**PBO (PD)–LAN (*N* = 10)**	**All patients (*N* = 41)**	**LAN (NPD)–LAN vs PBO (NPD)–LAN**	**LAN (NPD)–LAN vs PBO (PD)–LAN**
Clinical benefit rate[b]	21/21 (100)<br>[83.89; 100]	7/8 (87.5)<br>[47.35; 99.68]	5/9 (55.6)<br>[21.20; 86.30]	33/38 (86.8)<br>[71.91; 95.59]	12.50<br>[–28.37; 52.65]	44.44<br>[4.44; 78.80]

Note that objective response rate during the open-label treatment phase was not a pre-specified endpoint. Tumors were assessed according to RECIST v1.1 criteria.

[a]Complete responses plus partial responses.

[b]Complete responses plus partial responses plus stable disease.

CI, confidence interval; ITT, intention-to-treat; LAN, lanreotide autogel/depot; NPD, no progressive disease; PBO, placebo; PD, progressive disease; RECIST, response evaluation criteria in solid tumors.

Supplementary Table S9. Proportion of patients with a ≥10-point decrease (deterioration) in the EORTC QLQ-30 global health status scores during the DB phase (ITT population)

Time (weeks)	Proportion of patients (95% CI) [n/N]	
	LAN (*N* = 51)	Placebo (*N* = 26)
12	28.6 (15.7–44.6) [12/42]	21.7 (7.5–43.7) [5/23]
24	42.4 (25.5–60.8) [14/33]	29.4 (10.3–56.0) [5/17]
36	33.3 (18.0–51.8) [11/33]	50.0 (24.7–75.3) [8/16]
48	41.4 (23.5–61.1) [12/29]	25.0 (5.5–57.2) [3/12]
60	38.1 (18.1–61.6) [8/21]	33.3 (4.3–77.7) [2/6]
72	36.4 (10.9–69.2) [4/11]	33.3 (0.8–90.6) [1/3]
84	11.1 (0.3–48.2) [1/9]	0 (0–84.2) 0/2
96	0 (0–62.0) [0/4]	0
108	0 (0–97.5) [0/1]	0
Post-treatment	32.0 (14.9–53.5) [8/25]	66.7 (9.4–99.2) [2/3]

CI, confidence interval; DB, double-blind; EORTC, European Organisation for Research and Treatment of Cancer; ITT, intention-to-treat; LAN, lanreotide autogel/depot; QLQ-C30, Quality of Life Questionnaire Core 30

Supplementary Table S10. TGR[a] during the double-blind phase

Period[b]	Mean (95% CI) TGR[a] (% per month) [n]			
	Absolute		Change from baseline	
	LAN (*N* = 51)	Placebo (*N* = 26)	LAN (*N* = 51)	Placebo (*N* = 26)
Pre-baseline[c] to baseline	14.17 (3.86; 24.49) [15]	2.60 (–8.11; 13.32) [7]	–	–
Baseline to week 12	–0.11 (–4.30; 4.08) [46]	2.32 (–3.36; 7.99) [24]	–11.83 (–22.51; –1.15) [15]	11.00 (–3.99; 26.00) [7]
Weeks 12 to 24	–2.47 (–7.92; 2.97) [38]	3.21 (–0.95; 7.37) [20]	–12.47 (–26.94; 2.01) [11]	7.67 (–0.75; 16.09) [4]
Weeks 24 to 36	1.58 (–2.11; 5.28) [31]	3.26 (–1.13; 7.65) [18]	–13.88 (–30.94; 3.18) [9]	3.68 (0.99; 6.37) [3]
Weeks 36 to 48	–0.47 (–6.21; 5.28) [29]	2.16 (–2.39; 6.72) [12]	–4.40 (–10.87; 2.06) [8]	–3.89 (–142.77; 134.99) [2]

[a]TGR was calculated from radiological scans in the 12 months before the study, at baseline, and every 12 weeks during the study, and estimated using the sum of diameters of target lesions (100 (exp(3 log (Dt/D0)/t) – 1)) [D0 and Dt = tumor sizes at times 0 and t]; note that the provision of historical scans was optional, so TGR could only be computed for the subgroup of patients for whom historical scans were available; [b]TGR was assessed at 12-week intervals up to week 144, but patient numbers became increasingly small (20 [weeks 48–60] to 1 [weeks 96 to 108 onwards] in the LAN group and 7 to 1 in the placebo group), so data are not shown; [c]In the 12 months before baseline.

CI, confidence interval; LAN, lanreotide autogel/depot; TGR, tumor growth rate.

Supplementary Table S11. Most common[a] treatment-related TEAEs (safety and open-label ITT populations)

	***n* (%)**				
	**Double-blind phase**		**Open-label treatment phase**		
	**LAN group (*N* = 51)**	**PBO group (*N* = 26)**	**LAN (NPD)–LAN group (*N* = 21)**	**PBO (NPD)–LAN group (*N* = 9)**	**PBO (PD)–LAN group (*N* = 10)**
Diarrhea	27 (52.9)	4 (15.4)	1 (4.8)	1 (11.1)	3 (30.0)
Abdominal pain[b]	6 (11.8)	4 (15.4)	0	0	1 (10.0)
Fatigue	6 (11.8)	4 (15.4)	0	0	0
Flatulence	6 (11.8)	1 (3.8)	1 (4.8)	0	1 (10.0)
Abdominal distension	5 (9.8)	0	0	1 (11.1)	0
Nausea	5 (9.8)	1 (3.8)	0	0	0
Asthenia	4 (7.8)	1 (3.8)	0	0	1 (10.0)
Dizziness	4 (7.8)	0	0	0	0
Blood glucose increased	3 (5.9)	0	0	0	0
Injection-site reaction	3 (5.9)	0	0	0	0
Hyperglycemia	1 (2.0)	3 (11.5)	0	0	1 (10.0)
Anal incontinence	0	0	0	1 (11.1)	0

Median (range) duration of treatment exposure during the double-blind phase was 12.9 (1–33) months in the LAN group and 11.6 (1–24) months in the PBO group; during the open-label treatment phase, it was 7.5 (5–9) months in the LAN (NPD)–LAN group, 8.4 (3–12) months in the PBO (NPD)–LAN group, and 13.5 (1–21) months in the PBO (PD)–LAN group.

Data from the double-blind phase are from the safety population and those from the open-label treatment phase are from the open-label ITT population.

[a]Incidence ≥5% in any treatment group during the double-blind phase; [b]Preferred terms of abdominal pain or upper abdominal pain.

ITT, intention-to-treat; LAN, lanreotide autogel/depot; NPD, no progressive disease; PBO, placebo, PD, progressive disease; TEAE, treatment-emergent adverse event.

References

ECOG-ACRIN Cancer Research Group: ECOG Performance Status, 2020. https://ecog-acrin.org/resources/ecog-performance-status.

Eisenhauer, EA, Therasse, P, Bogaerts, J, Schwartz, LH, Sargent, D, Ford, R, Dancey, J, Arbuck, S, Gwyther, S, Mooney, M, Rubinstein, L, Shankar, L, Dodd, L, Kaplan, R, Lacombe, D & Verweij, J 2009. New response evaluation criteria in solid tumours: revised RECIST guideline (version 1.1). *Eur J Cancer,* 45**,** 228-47.
